# New Polyketides from Mangrove Endophytic Fungus *Penicillium* sp. BJR-P2 and Their Anti-Inflammatory Activity

**DOI:** 10.3390/md20090583

**Published:** 2022-09-18

**Authors:** Chen Chen, Geting Ye, Jing Tang, Jialin Li, Wenbin Liu, Li Wu, Yuhua Long

**Affiliations:** GDMPA Key Laboratory for Process Control and Quality Evaluation of Chiral Pharmaceuticals, Guangzhou Key Laboratory of Analytical Chemistry for Biomedicine, School of Chemistry, South China Normal University, Guangzhou 510006, China

**Keywords:** isocoumarins, polyketides, anti-inflammatory activity, *Penicillium* sp.

## Abstract

Four new polyketide compounds, including two new unique isocoumarins penicillol A (**1**) and penicillol B (**2**) featuring with spiroketal rings, two new citreoviridin derivatives citreoviridin H (**3**) and citreoviridin I (**4**), along with four known analogues were isolated from the mangrove endophytic fungus *Penicillium* sp. BJR-P2. Their structures were elucidated by extensive spectroscopic methods. The absolute configurations of compounds **1**–**4** based on electronic circular dichroism (ECD) calculations, DP4+ analysis, and single-crystal X-ray diffraction are presented. All the new compounds were evaluated for anti-inflammatory activity. An anti-inflammatory assay indicated that compound **2** inhibited lipopolysaccharide (LPS)-induced NO production in RAW 264.7 cells, with half-maximal inhibitory concentration (IC_50_) values of 12 μM, being more potent than the positive control, indomethacin (IC_50_ = 35.8 ± 5.7 μM). Docking study showed that compound **2** was perfectly docking into the active site of murine inducible nitric oxide oxygenase (iNOS) via forming multiple typical hydrogen bonds.

## 1. Introduction

Inflammation is an adaptive response triggered by harmful stimuli, including some conditions of infection and tissue damage [1,2]. Macrophages, neutrophils, and lymphocytes are important natural immune cells to participate in homeostasis and immune response, and play complicated action on the pathogenesis of inflammatory disease [3,4,5]. Stimulated immune cells regulate inflammation by producing proinflammatory factors and mediators, such as interleukin (ILS), tumor necrosis factor-α (TNF-α), NO, prostaglandin E2 (PGE2), and iNOS [6,7,8]. Therefore, inhibition of inflammatory cytokines, chemokines, and mediators can be potent therapeutic strategies for the prevention of inflammation-related diseases.

**Figure 1:**
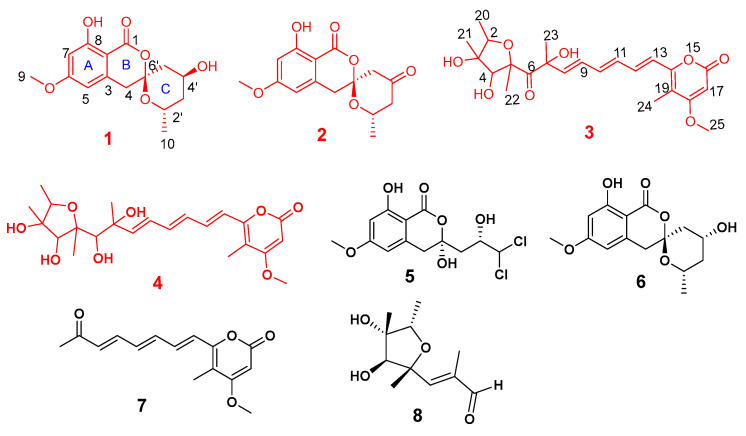
Chemical structures of compounds **1**–**8**.

*Penicillium* species are among the most widespread fungal organisms on earth and contains more than 350 species. Many *Penicillium* species can produce plentiful secondary metabolites, such as alkaloids [9], polyketides [10], cyclic peptides [11], and terpenoids [12], that can ascribe specific structural characteristics and significant biological activities. Isocoumarins are important natural lactones with wide range of biological activities, such as neuroprotective, antibacterial, antivirus and antitumor activities, and distribute widely in various microorganisms and plants from natural sources [13,14,15,16,17,18,19]. Up to now, nearly 1000 naturally occurring isocoumarins were reported [14]. As our continuing interest in finding new compounds with potential anti-inflammatory activity, the chemical investigation of the endophytic fungus *Penicillium* sp. BJR-P2 yielded two new unique isocoumarins with spiroketal rings (**1**–**2**), two new citreoviridin derivatives (**3**–**4**), together with four known analogues (**5**–**8**) (Figure 1). In this paper, the stereochemistry of these compounds was determined for the first time by DP4+ analysis, ECD calculations, and single-crystal X-ray diffraction. By screening of the inhibitory effects on NO production in the LPS-induced RAW 264.7 macrophages, the anti-inflammatory activities of these compounds were evaluated, and the results showed that compound **2** exhibited effective inhibitory activity with IC_50_ value of 12 μM. This article describes the isolation, structure elucidation, and NO production inhibition of the new compounds.

## 2. Results and Discussion

### 2.1. Identification and Purification

The EtOAc extract of marine-derived fungus Penicillium sp. BJR-P2 was performed on repeated silica gel and Sephadex LH-20 column chromatography, followed by semipreparative HPLC to afford four new polyketides, penicillol A (**1**), penicillol B (**2**), and citreoviridin H (**3**), and citreoviridin I (**4**), along with four known polyketides **5**, **6**, **7,** and **8**.

### 2.2. Structural Elucidation

**Figure 2:**
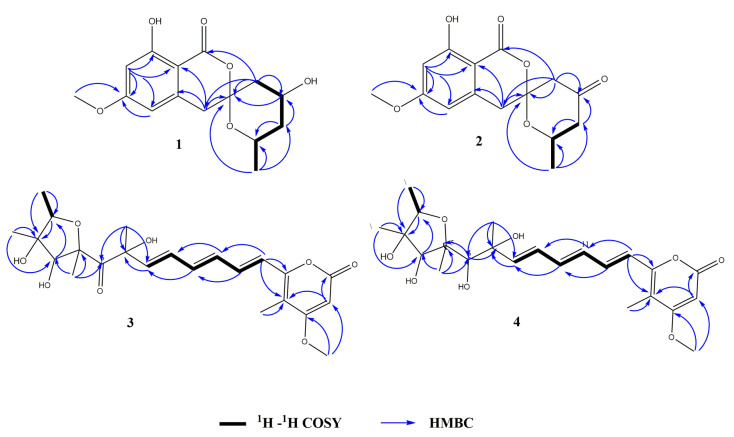
Key HMBC and ^1^H-^1^H COSY correlations of **1**–**4**.

**Figure 3:**
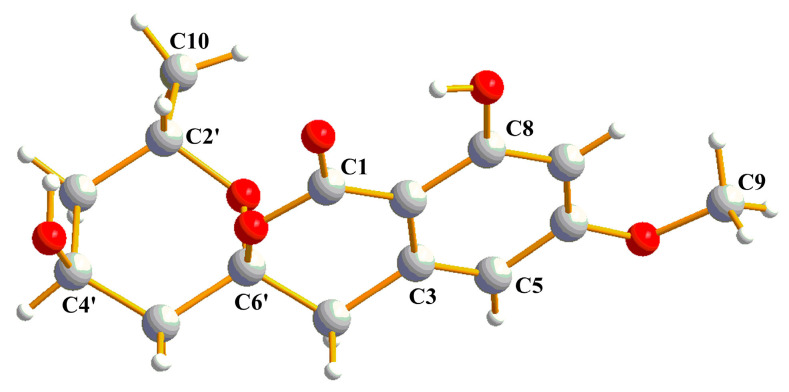
ORTEP representation of crystal structure of **1**.

Compound **1** was obtained as a white powder. Its molecular formula was determined to be C_15_H_1__7_O_6_ from the HRESIMS (*m/z* 293.1024 [M − H]^−^ calcd for C_15_H_1__7_O_6_, 293.1031), indicating 7 degrees of unsaturation. The IR spectrum of **1** at 3696, 1646, and 1585 cm^−1^ suggested the presence of hydroxyl, carbonyl, and aromatic ring groups. The ^13^C NMR data in combination with HMQC spectra (Table 1) displayed one methyl carbon at *δ*_C_ 20.5, one methoxyl carbon at *δ*_C_ 55.2, three methylene carbons at *δ*_C_ 39.1, 38.9, and 38.5, four methine carbons at *δ*_C_ 106.8, 99.3, 63.4, 63.0, and six nonprotonated carbons at *δ*_C_ 169.2, 166.7, 164.4, 140.2, 104.6, 101.0. Analysis of the ^1^H NMR spectrum of **1** revealed two aromatic protons at *δ*_H_ 6.35 (d, *J* = 2.1), 6.34 (s), indicating the presence of a tetrasubstituted phenyl. Two oxygenated methine protons signal at *δ*_H_ 4.39 (m) and 4.17 (m), three aliphatic methylenes at *δ*_H_ 1.53 (m), 1.78 (m), 1.87 (dd, *J* = 4.08, 15 Hz), 2.21 (dt, *J* = 15, 2.2 Hz), 2.95 (d, *J* = 16.5 Hz), and 3.17 (d, *J* = 16.5 Hz), one methoxyl at *δ*_H_ 3.82 (s), and one methyl at *δ*_H_ 1.07 (d, *J* = 6.3 Hz) were also recorded in this spectrum. The above spectroscopic features indicated that **1** belonged to the isocoumarin class. Further analysis of HMBC spectrum (Figure 2), the correlations from H-7 to C-2 (*δ*_C_ 101.0)/C-5 (*δ*_C_ 106.8)/C-6 (*δ*_C_ 166.7)/C-8 (*δ*_C_ 164.4), from H-5 to C-2/C-6, from H-4 to C-2/C-3 (*δ*_C_ 140.2)/C-6’ (*δ*_C_ 104.6), from H-9 to C-6 suggested that **1** was an isocoumarin derivative with a hydroxyl group at C-8 and a methoxy group at C-6. The ^1^H-^1^H COSY correlations between H-2’, H-3’, H-4’, H-5’, and H-10 combined with the HMBC correlations from H-5’ to C-3’ (*δ*_C_ 38.5)/C-4’ (*δ*_C_ 63.4), from H-3’ to C-2’ (*δ*_C_ 63.0)/C-10 (*δ*_C_ 20.5), from H-10 to C-2’/C-3’ suggested an aliphatic fragment of -CH_2_-CH-CH_2_-CH-CH_3_. Furthermore, the key HMBC correlations from H-5’ to C-1/C-4/C-6’, from H-4’ to C-6’, and from H-10 to C-6’, together with the unsaturation of compound **1** and the chemical shift of C6’ (*δ*_C_ 104.6) indicated the presence of the spiroketal ring C. Therefore, the planar structure of **1** was shown in Figure 1. The absolute configuration of **1** was further verified by the X-ray diffraction analysis of a single crystal using Cu K*α* as 6’*S*, 2’*S*, 4’*S*-**1** (Figure 3). Hence, the structure of compound **1** was identified as 6′*S*, 2’*S*, 4′*S*-**1** and named penicillol A.

**Figure 4:**
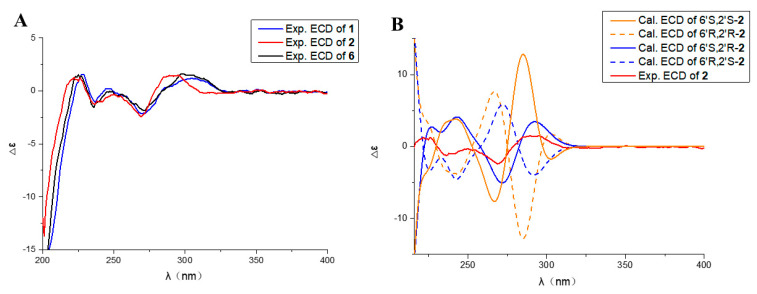
(**A**) Experimental ECD spectra of compounds **1**, **2,** and **6**. (**B**) Comparison between the experimental and calculated ECD spectra of **2**.

Compound **2,** white powder, possesses the molecular formula C_15_H_15_O_6_, as assigned by the HRESIMS ion at *m/z* 291.08727 [M − H]^−^ (calcd for C_15_H_15_O_6_, 291.08741), showing eight degrees of unsaturation. The IR spectrum suggested the presence of hydroxy (3680 cm^−1^), aromatic ring (1580 cm^−1^), and carbonyl (1736 cm^−1^, 1671 cm^−1^). Analysis of the ^13^C NMR and HMQC (Table 1) data indicated the presence of 15 carbon atoms consisting of one methoxy group, one methyl, three methylenes, three methines (including two aromatic methines), 7 nonprotonated carbons (including one ester carbon, one carbonyl, four olefinic carbons). The ^1^H NMR spectrum of **2** displayed two aromatic protons at *δ*_H_ 6.38 (d, *J* = 2.3 Hz), 6.29 (s), indicating the presence of a tetrasubstituted phenyl. One oxygenated methine proton signal at *δ*_H_ 4.41 (m), three aliphatic methylenes at *δ*_H_ 3.14 (d, *J* = 16.4), 3.20 d, *J* = 16.4 Hz), 2.29 (dd, *J* = 11.5, 15.1 Hz), 2.53 (m), 2.59 (d, *J* = 15.4 Hz), and 2.85 (dd, *J* = 1.6, 15.3 Hz), one methoxyl at *δ*_H_ 3.84 (s), one methyl at *δ*_H_ 1.24 (d, *J* = 6.2 Hz), and one active proton at *δ*_H_ 11.04 (s) were also recorded in this spectrum. Cumulative analyses of the ^1^H and ^13^C NMR spectra of compound **2** revealed that it possessed the similar planar structure as that of **1**. The main difference was the change of hydroxylated methine in **1** (*δ*_C_ 63.4) to a carbonyl in **2** (*δ*_C_ 202.5), indicating that compound **2** was an analog of **1**. Furthermore, the planar structure of **2** was further verified by the key HMBC correlation from H-5’ to C-4’ (*δ*_C_ 202.5), and H-3’ to C-4’ (Figure 2). The absolute configuration of **2** was determined by comparison of experimental and calculated ECD. Experimental data showed that the compounds **1**, **2**, and **6** had extremely similar ECD spectra, inferring that the cotton effect of compound **2** might be only affected by the chiral centers of C-6’ (*δ*_C_ 104.7) and C-2’ (*δ*_C_ 67.9) (Figure 4A). Then, the ECD spectra of the four possible configurations (6’*S*, 2’*S*-**2**; 6’*S*, 2’*R*-**2**; 6’*R*, 2’*S*-**2**; 6’*R*, 2’*R*-**2**) were calculated. The results disclosed that 6’*S*, 2’*S*-**2** and 6’*S*, 2’*R*-**2** displayed similar ECD spectra with the experimental one, showing a negative cotton effect (CE) at about 266 nm and a positive CE at about 285 nm (Figure 4B). Therefore, it was rationally speculated that the cotton effect of compound **2** was only affected by the chiral center of C-6’, and the configuration of C-6’ was determined to be 6’S as same as that of compound **1**. Based on the biosynthetic point of view, the absolute configuration at C- 2′ should be the same as that of compound **1**. Therefore, the structure of compound **2** was identified as 6′*S*, 2’*S*-**2** and named as penicillol B.

**Figure 5:**
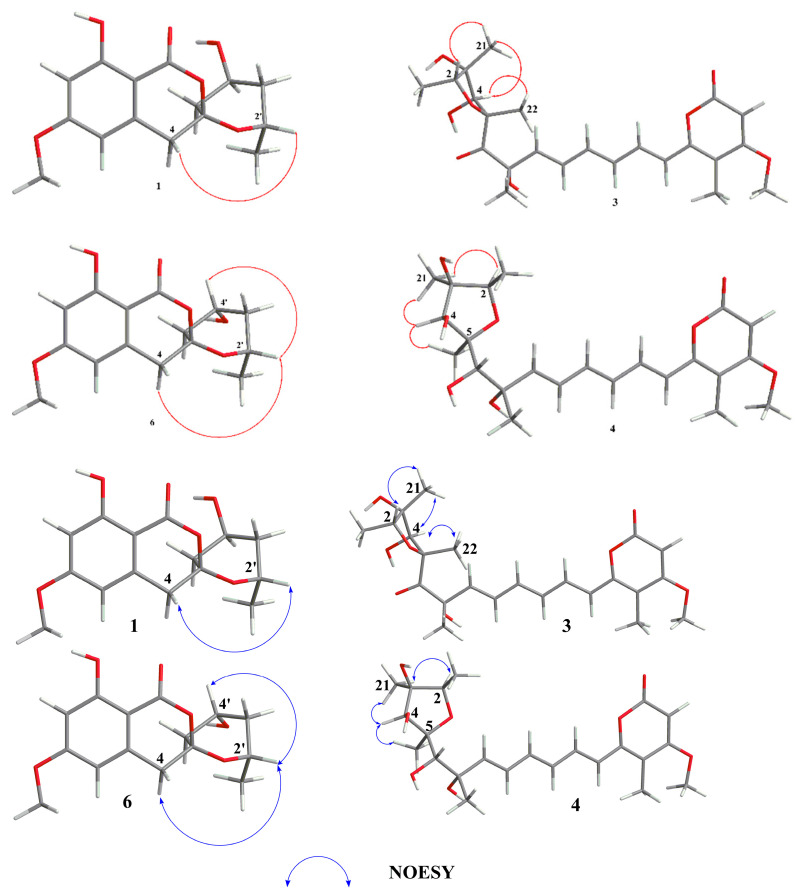
Key NOESY correlations of **1**, **3**, **4,** and **6**.

Compound **3** was obtained as pale-yellow oil with the molecular formula C_23_H_30_O_8_ established from HRESIMS at *m/z* 415.17607 [M − H_3_O]^−^ (calcd for 415.17623), showing 9 degrees of unsaturation. The IR spectrum at ν_max_ 3685, 1725, 1693, and 1612 cm^−1^, corresponded to a hydroxy, ester carbonyl, carbonyl and double bond group, respectively. Analysis of the ^13^C NMR and HMQC spectra revealed 23 carbon resonances, corresponding to one ketone carbonyl (*δ*_C_ 204.7), one ester carbonyls (*δ*_C_ 163.9), three sp^2^ nonprotonated carbons (*δ*_C_ 108.2, 154.5 and 170.8), three sp^3^ nonprotonated carbon (*δ*_C_ 86.3, 84.9 and 82.3), seven sp^2^ methine carbons (*δ*_C_ 88.9, −139.1), two sp^3^ methine carbons (*δ*_C_ 80.2, 78.1), and five methyl carbons (*δ*_C_ 31.4, 16.9, 13.1, 12.5 and 9.0), one methoxyl (*δ*_C_ 56.3). Analysis of ^1^H NMR spectrum of **3** exhibited a set of olefinic protons resonances at *δ*_H_ 5.49 (s), 6.34 (d, *J* = 15.0 Hz), 6.27 (dd, *J* = 15.5, 10.5 Hz), 6.36 (dd, *J* = 15.0, 10.5 Hz), 7.16 (dd, *J* = 15.0, 10.5 Hz), 6.43 (dd, *J* = 15.0, 10.5 Hz), 6.05 (d, *J* = 15.5 Hz,). Five methyls at *δ*_H_ 1.96 (s), 1.39 (s), 1.23 (d, *J* = 6.5 Hz), 1.35 (s), 1.46 (s), one methoxyl at *δ*_H_ 3.82 (s), and two oxygenated methine protons signal at *δ*_H_ 3.94 (s), 4.24 (dd, *J* = 6.5, 12.9 Hz) were also recorded. All the above data were indicative that compound **3** possessed a similar carbon skeleton as that of citreoviridin which was a polyketide derivative from a *Penicillium pulvillorum* [20]. The HMBC correlations from H-20 to C-2 (*δ*_C_ 80.2)/C-3 (*δ*_C_ 86.3), from H-2 to C-3, from H-21 to C-3/C-4 (*δ*_C_ 78.1), from H-4 to C-2, and from H-22 to C-4/C-5 (*δ*_C_ 84.9) suggested the presence of **3**, 4-dihydroxy-2, 3, 5-trimethyl-tetrahydrofuran moiety (Figure 2). The ^1^H-^1^H COSY correlation of H-2/ H_3_-20 and H-8/ H-9/ H-10/ H-11/ H-12/ H-13 indicated two hydrocarbon fragments of -CH-CH_3_ and -CH=CH-CH=CH-CH=CH-. Through the analysis of the ^1^H NMR spectra of **3** to those of citreoviridin, combined with key HMBC correlation from H-17 to C-16/C-18/C-19, from H-24 to C-19, and from H-25 to C-17/C-18, suggested the presence of 4-methyl-5-methoxy-3, 4, 5-trisubstituted-α-pyrone moiety. Moreover, the HMBC correlation from H-13 to C-14 (*δ*_C_ 154.5)/C-19 (*δ*_C_ 108.2), indicated the unsaturated hydrocarbon fragment was lactated at C-14 of α-pyrone moiety. The main difference was the replacement of two double bond carbons in citreoviridin by a carbonyl carbon C-6 (*δ*_C_ 204.7) and an oxygenated quaternary carbon C-7 (*δ*_C_ 82.3) in **3**. This deduction was supported by the key HMBC correlations from H-23 to C-7/C-6 (*δ*_C_ 204.7)/C-8 (*δ*_C_ 139.1), from H-8 to C-7 (*δ*_C_ 82.3), and from H-22 to C-5/C-6. So, the planar structure of **3** was elucidated, as shown in Figure 1. The NOE correlations from H-2 to Me-21, from Me-21 to H-2/H-4, from Me-22 to H-4 revealed the relative configuration of the five-membered ring as 2R*, 3S*, 4S*, 5R* (Figure 5). The relative configuration of C-7 was subjected to DP4+ analysis. Therefore, a DP4+ analysis of two candidate structures (2R*, 3S*, 4S*, 5R*, 7S*-**3**/2R*, 3S*, 4S*, 5R*, 7R*-**3**) was performed by calculating for their theoretical 1D NMR chemical shifts. The result showed that the final score of the configuration 2R*, 3S*, 4S*, 5R*, 7S*-**3** (100%) was the most probable (Figures S29 and S30). Thus, the structure of compound **3** was identified as 2R*, 3S*, 4S*, 5R*, 7S*-**3**. Coupling constants between protons H-8 and H-9, H-10 and H-11, H-12 and H-13 (^3^*J*_H-8, H-9_ = 15.5 Hz; ^3^*J*_H-10, H-11_ = 15.0 Hz; ^3^*J*_H-12_, _H-13_ = 15.0 Hz) inferred that the conformations of these three double bonds are all *trans* conformations, and compound **3** is named citreoviridin H.

Compound **4** was isolated as pale-yellow oil, and its molecular formula was determined as C_23_H_32_O_8_ on the basis of the pseudomolecular ion peak observed at *m/z* 417.19163 [M − H_3_O]^−^ (Calcd for 417.19188) in the HRESIMS spectrum, indicating 8 degrees of unsaturation. The IR spectrum showed absorption bands of hydroxyl, carbonyl, and double bond at νmax 3696, 1694, and 1623 cm^−^^1^. The ^13^C NMR and HMQC spectra (Table 2) displayed 23 carbon signals, consisting of six methyls (including one methoxyl), ten methines (including seven olefinic carbons), and seven quaternary carbons (including one ester carbon). The ^1^H NMR spectrum of **4** revealed a set of olefinic protons resonances at *δ*_H_ 5.64 (s), 6.59 (d, *J* = 15.0 Hz), 6.43 (dd, *J* = 11.0, 15.4 Hz), 6.5 (dd, *J* = 11.0, 15.0 Hz), 7.16 (dd, *J* = 11.0, 15.0 Hz), 6.65 (dd, *J* = 11.0, 15.0 Hz), 6.01 (d, *J* = 15.4 Hz), five methyls at *δ*_H_ 2.02 (s), 1.32 (s), 1.20 (d, *J* = 6.4 Hz), 1.22 (s), 1.30 (s), one methoxyl at *δ*_H_ 3.92 (s), and three oxygenated methine protons signal at *δ*_H_ 3.79 (s), 4.08 (m), 3.69 (s). All the above data were indicative that compound **4** possessed a similar carbon skeleton of **3**. The main difference was the replacement of a carbonyl group in **3** (*δ*_C_ 204.7) by a hydroxymethine group in **4** (*δ*_C_ 91.2), indicating that compound **4** was a homologue of **3**. This inference was further confirmed by the key HMBC correlation from H-22 to C-5 (*δ*_C_ 86.3)/C-6 (*δ*_C_ 91.2), from H-6 to C-5/C-7 (*δ*_C_ 73.3)/C-8 (*δ*_C_ 140.9), and from H-23 to C-7/C-6 (Figure 2). Therefore, the planar structure of **4** was established as shown in Figure 1. Furthermore, the relative configuration of compound **4** was partly determined by NOESY spectrum. The NOE correlations from H-2 to Me-21, from Me-21 to H-2/H-4, from Me-22 to H-4 indicated the relative configuration of the tetrahydrofuran ring to be 2R*, 3S*, 4S*, 5S* (Figure 5). In order to determine the relative configuration of C-6 and C-7, the DP4+ analysis of four candidate structures (2R*, 3S*, 4S*, 5S*, 6S*, 7S*-**4**/2R*, 3S*, 4S*, 5S*, 6R*, 7S*-**4**/2R*, 3S*, 4S*, 5S*, 6R*, 7R*-**4**/2R*, 3S*, 4S*, 5S*, 6S*, 7R*-**4**) was performed. The analysis results showed that the configuration of 2R*, 3S*, 4S*, 5S*, 6S*, 7S*-**4** was the correct structure with a 100% probability (Figures S31 and S32). Hence, the structure of compound **4** was identified as 2R*, 3S*, 4S*, 5S*, 6S*, 7S*-**4**. Coupling constants between protons H-8 and H-9, H-10 and H-11, H-12 and H-13 (^3^*J*_H-8, H-9_ = 15.4 Hz; ^3^*J*_H-10, H-11_= 15.0 Hz; ^3^*J*_H-12, H-13_ = 15.0 Hz) inferred that the conformations of these three double bonds are all *trans* conformations, and compound **4** is named citreoviridin I.

Four known compounds including dichlorodiaportal (**5**) [21], citreoviranol (**6**) [22], citreopyrone D (**7**) [23], citreoviral (**8**) [24], were isolated and identified from this fungus. Their structures were determined by comparing their NMR and MS data with those reported in the literature. Moreover, the absolute configuration of compound **6** was further determined to be 6’*S*, 2’*S*, 4’*R*-**6** by comparing its NOE correlations and ECD spectrum with compound **1.**

A plausible typical fungal polyketide synthetase (PKS) involved biosynthetic pathway for compounds **1**–**4** was proposed as shown in Figure S33. The condensation of one mole of acetyl coenzyme A and six moles of malonyl coenzyme A gives a mole linear polyketide chain. Subsequent keto-reduction, cyclization, methylation, and hydroxylation furnish compounds **1** and **2.** Similarly, one mole acetyl coenzyme A and eight moles malonyl coenzyme A condensed to form a mole linear polyketide chain, then keto-reduction, dehydration, cyclization, methylation, and hydroxylation form compounds **3** and **4** [25,26].

### 2.3. Anti-Inflammatory Activity

New compounds **1**–**4** were evaluated for anti-inflammatory activity in LPS-stimulated RAW 264.7 macrophages. Especially, Compound **2** significantly inhibited nitric oxide production with an IC_50_ value of 12 μΜ (Table 3).

### 2.4. Molecular Docking Studies

**Figure 6:**
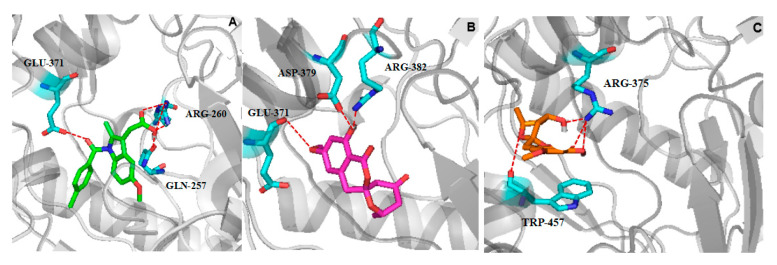
(**A**) Docking results of the binding pose of positive drug indomethacin in iNOS. (**B**) Predicted binding mode of compound **2** docked into iNOS (PDB: 3E6T). (**C**) Predicted binding mode of compound **1** docked into iNOS. (PDB: 3E6T) (indomethacin is in green stick; compound **2** is in purple stick; compound **1** is in yellow stick; red dashed lines represent H-bonds; the amino acids involved in hydrogen bond interactions are in blue stick).

Inhibition of NO overproduction is usually the result of inhibition of iNOS enzyme expression or activity [27,28]. In order to investigate the inhibitory mechanism of compound **2** on NO production, the interaction and binding mode between compound **2** and iNOS (PDB: 3E6T) [29], molecular docking study was carried out using AUTODOCK 4.2.6 modeling software. Docking procedure was validated by docking of ligand indomethacin (positive drug) in the active site of iNOS, and root-mean square deviation (RMSD) of 0.12 Å to the X-ray structure. The results revealed that the lowest energy of compound **2** (−7.49 Kcal/mol) was lower than that of positive drug indomethacin (−7.45 Kcal/mol), and the lowest energy of compound **1** (−7.36 Kcal/mol) was higher than that of positive drug. Further observations showed indomethacin formed a hydrogen bond with key amino acid residues GLU-371, two hydrogen bonds with amino acid residues ARG-260, and a hydrogen bond with GLN-257 in the iNOS active pocket (Figure 6A). Compound **2** formed a hydrogen bond with the key amino acid residue GLU-371 through the methoxy group, two hydrogen bonds with the residue ASP-379 and ARG-382 by the hydroxyl group in the iNOS active pocket (Figure 6B), respectively. Notably, for compound **1**, while the carbonyl group in compound **2** at the 4’ position changed to the hydroxyl group, the optimized conformation of **1** was different from that of compound **2** and could not enter into the iNOS active pocket to form hydrogen bonds with the key amino acid residues (Figure 6C). As a result, compound **1** had no production inhibition activity (Table 3).

## 3. Materials and Methods

### 3.1. General Experimental Procedures

HRESIMS data were measured on a Finnigan LTQ-Orbitrap Elite (Thermo Fisher Scientific, Waltham, MA, USA). NMR spectra were obtained by Bruker AVANCE NEO 600 MHz spectrometer (Bruker BioSpin, Switzerland). CD spectrum was reported on a Chirascan TM CD spectropolarimeter (Applied Photophysics, U.K). Optical rotation was obtained on an MCP 500 (Anton Paar, Austria). Single-crystal data was measured on an Oxford Gemini S Ultra diffractometer (Oxford Instrument, Oxfordshire, UK). Sephadex LH-20 (25–100 μm; GE Healthcare, Bio-Sciences AB, Stockholm, Sweden) and silica gel (200–300 mesh; Qingdao Marine Chemical Factory, Qingdao, China) were used for Column chromatography (CC). Thin layer chromatography (TLC) was detected on Silica gel GF254 plate (Qingdao Marine Chemical Ltd., Qingdao, China).

### 3.2. Fungal Material

The fungus BJR-P2 used in this research was isolated from the barks of *Avicennia marinav* (*Forsk*.) *Vierh*, a mangrove plant which collected from Yangjiang Hailing Island Mangrove Wetland Park. Using molecular biology methods, the fungus was identified through DNA amplification and ITS region sequencing. The sequence data of this strain has been deposited in Gen Bank with accession no. PRJNA793386. The BLAST search results show that the sequence is 100% similar to that of *Penicillium* sp.

### 3.3. Extraction and Isolation

The fungus *Penicillium* sp. BJR-P2 was fermented on solid autoclaved rice medium using one hundred 1 L Erlenmeyer flasks, each was containing 40 g rice and 40 mL 0.3% saline water, culturing in room temperature under static condition for 25 days. The solid rice and mycelia medium were extracted with methanol for three times. The organic solvents were evaporated under reduced pressure and extracted with ethyl acetate. We obtained 50 g of organic extract. Then, the extract was subjected to a silica gel column eluting with a gradient of petroleum ether/EtOAc from 1/0 to 0/1 to afford six fractions (Fractions 1–6). Fraction 2 (300 mg) was subjected to an open silica gel glass column eluted with DCM:MeOH (100:1) followed by Sephadex LH-20 open glass (30 mg) CC eluting with MeOH-H_2_O (*v/v*, 7:3), and further purified by using HPLC on a semipreparative column (RP-18, 9.4 × 250 mm, 7 μm, 1.5 mL min^−1^) eluted with MeOH:H_2_O (6:4) to obtain compounds **1** (9.0 mg) and **4** (5.0 mg). Further separation of fraction 3 by preparative HPLC over silica gel (250 mm × 10 mm) with PE-EtOAc (4:2–1:1) and DCM:MO (200:1-80:1) as the eluent afforded **2** (10.0 mg) and **3** (5.0 mg). Fraction 4 (500 mg) was chromatographed on Sephadex LH-20 CC and silica gel CC to obtain compounds **5–8**.

Compound **1**: white powder; ${\left[\alpha \right]}_{D}^{25}-88.9{}^{\circ}\;\left(c\;0.1,\;\mathrm{MeOH}\right)$; IR (KBr): ν_max_ 3696, 1646, 1585 cm^−^^1^; UV (MeOH) λ_max_ (logε): 216 (0.91) nm; 268 (0.53) nm; 303 (0.22); ECD (MeOH) ***λ***_max_ (Δε), 228 (+1.56), 237 (−1.01), 247 (+2.02), 270 (−2.15), 305 (+1.20) nm; HRESIMS at *m/z* 293.1024 [M − H]^−^ (calcd for 293.1031). ^1^H and ^13^C NMR see Table 1.

Compound **2**: white powder; ${\left[\alpha \right]}_{D}^{25}-62.0{}^{\circ}\;\left(c\;0.1,\;\mathrm{MeOH}\right)$; IR (KBr): ν_max_ 3680, 1736, 1671, 1580 cm^−^^1^; UV (MeOH) λ_max_ (log ε): 216 (0.89) nm; 268 (0.52) nm; 303 (0.21); ECD (MeOH) λ_max_ (Δε), 222 (+1.25), 236 (−1.23), 250 (−0.31), 269 (−2.43), 295 (+1.48) nm; HRESIMS at *m/z* 291.08727 [M − H]^−^ (calcd for 291.08741). ^1^H and ^13^C NMR see Table 1.

Compound **3**: yellow oil; ${\left[\alpha \right]}_{D}^{25}-10{}^{\circ}\;\left(c\;0.1,\;\mathrm{MeOH}\right)$; IR (KBr): ν_max_ 3685, 1725, 1693, 1612 cm^−^^1^; UV (MeOH) λ_max_ (logε): 274 (0.65) nm; 367 (0.5) nm; HRESIMS at *m/z* 415.17607 [M − H_3_O]^−^ (calcd for 415.17623). ^1^H and ^13^C NMR see Table 2.

Compound **4**: yellow oil; ${\left[\alpha \right]}_{D}^{25}-10{}^{\circ}\;\left(c\;0.1,\;\mathrm{MeOH}\right)$; IR (KBr): ν_max_ 3696, 1694, 1623 cm^−^^1^; UV (MeOH) λ_max_ (logε): 274 (0.96) nm; 368 (0.78) nm; HRESIMS at *m/z* 417.19163 [M − H_3_O]^−^ (calcd for 417.19188). ^1^H and ^13^C NMR see Table 2.

X-ray Crystal Data for **1**. Colorless crystal of **1** was obtained in methanol and EtOAc. Crystal data (CCDC 2131096) were collected with Cu Kα radiation. Monoclinic, space group P21, a = 7.18058(6) Å, b = 7.48777 (11) Å, c = 13.48676 (10) Å, α = 90°, β = 92.3992 (7) °, γ = 90, V = 724.501(14) Å3, Z = 2, T = 149.99(10) K, μ (Cu Kα) = 0.963 mm^−^^1^, ρcalc = 1.432 g/cm^3^, F (000) = 332.0, R_1_ = 0.0296, wR_2_ =0.0745. Crystal dimensions 0.28 × 0.16 × 0.12 mm^3^. Flack parameter = 0.03(4). The total number of independent reflections measured was 12,512, of which 2626 were observed, collected in the range of 6.56° ≤ 2θ ≤ 145.682°. The structure was determined and refined using full-matrix least-squares on F2 values for 1.109 I > = 2σ (I).

### 3.4. ECD and ^13^C NMR Calculation

The conformational searches of the compounds were carried out by means of the Spartan’14 software and at Molecular Merck force field (MMFF) and DFT/TD-DFT calculations. Furthermore, Gaussian 05 program was used to generate and optimize the conformer at B3LYP/3-21G (d) level. Conformers with a Boltzmann distribution of over 1% were chosen for optimization at B3LYP/6-31+G (d, p), meanwhile, ECD calculation were conducted with the TD-DFT method at the B3LYP/6-31+G (d, p) level and the ^13^C NMR calculation at mPW1PW91-SCRF/6-311+g (2d, p). The ECD spectra were generated using the SpecDis 3.0 (University of Würzburg, Würzburg, Germany) and Origin Pro 8.0 (Origin Lab, Ltd., Northampton, MA, USA) from dipole length rotational strengths by applying Gaussian band shapes with sigma = 0.30 eV. Then, the calculated and theoretical values of ^13^C were analyzed by DP4+ [30].

### 3.5. Anti-Inflammatory Assays

The RAW 264.7 mouse macrophage cell line was purchased from the Cell Bank of Shanghai Institute of Biochemistry and Cell Biology (Chinese Academy of Sciences, Shanghai, China). Murine macrophage RAW 264.7 cells were cultured in DMEM (high glucose) medium supplemented with 10% (*v/v*) fetal bovine serum, 100 µg·mL^−1^ penicillin and streptomycin, and 10 mM HEPES buffer at 37◦C in 5% CO_2_ in air for 1 h. Cells were pretreated with different concentrations of samples (10, 5, 2.5, 1.25, and 0.625 µM) dissolved in serum-free medium containing 0.5% DMSO for 4 h, followed by stimulation with 1 µg·mL^−1^ LPS for 24 h. A total of 50 µL cell culture medium was mixed with 100 µL Griess reagents I and II and incubated horizontally at room temperature for 10 min. The absorbance was measured at 570 nm [31,32].

### 3.6. Molecular Docking Study

Virtual docking is implemented in the AutoDock tool of AutoDock4.2.6 software [33]. This is a common docking method that allows the ligand to have sufficient flexibility and maintain the rigidity of the target protein. The X-ray crystal structure of iNOS (PDB ID: 3E6T) [27] was obtained from the RCSB protein database (PDB) database. Before docking simulation, PyMOL was used to delete the original ligand and water molecules from the crystal structure, and the protein was saved in PDB format (receptor.pdb). The compound structure was drawn using ChemDraw 2D software, which was converted into three-dimensional (3D) structure by ChemDraw 3D software, and then stored as a file in PDB format. Furthermore, the molecular structure was optimized by Gaussian software. AutoDock tools converted both protein and ligand into PDBQT format for subsequent docking. Focusing on the protein, the parameters of the grid box were set to 126 × 126 × 126 points and the Lamarckian genetic algorithm was used to link the algorithm with 100 GA operations. Finally, PyMOL was used to visualize and analyze the results.

## 4. Conclusions

In conclusion, chemical investigation of the mangrove endophytic fungus BJR-P2 resulted in the isolation and identification of four new compounds (**1**–**4**), with four known analogs (**5**–**8**). Their structures were elucidated by extensive spectroscopic methods and quantum chemical calculations. The anti-inflammatory activity evaluation was carried out by screening their inhibition activity on NO production. The results showed compound **2** exhibited significant inhibitory activity with an IC_50_ value of 12 µM. This study may provide a new chemical lead candidate for the discovery of anti-inflammatory agents.

## Figures and Tables

**Table 1:** ^1^H (600 MHz) and ^13^C NMR (150 MHz) data for compounds **1** and **2**.

Position	1 ^a^ (*δ*_C_, Type)	1 ^a^ (*δ*_H_, Type)	2 ^b^ (*δ*_C_, Type)	2 ^b^ (*δ*_H_, Type)
1	169.2, C		167.5, C	
2	101.0, C		100.6, C	
3	140.2, C		138.3, C	
4	39.1, CH_2_	2.95, d (16.5)	38.6, CH_2_	3.14, d (16.4)
		3.17, d (16.5)		3.20, d (16.4)
5	106.8, CH	6.34, s	107.4, CH	6.29, s
6	166.7, C		166.4, C	
7	99.3, CH	6.35, d (2.1)	99.7, CH	6.38, d (2.3)
8	164.4, C		164.7, C	
9	55.2, CH_3_	3.82, s	55.8, CH_3_	3.84, s
10	20.5, CH_3_	1.07, d (6.3)	21.6, CH_3_	1.24, d (6.2)
2′	63.0, CH	4.39, m	67.9, CH	4.41, m
3′	38.5, CH_2_	1.53, m	47.9, CH_2_	2.29, dd (11.5, 15.1)
		1.78, m		2.53, m
4′	63.4, CH	4.17, m	202.5, C	
5′	38.9, CH_2_	1.87, dd (4.1, 15.0)	49.5, CH_2_	2.59, d (15.4)
		2.21, dt (2.2, 15.0)		2.85, dd (1.56, 15.3)
6′	104.6, C		104.7, C	
8-OH				11.0, s

^a^ Measure in MeOD-d_4_. ^b^ measure in CDCl_3_.

**Table 2:** ^1^H (600 MHz) and ^13^C NMR (150 MHz) data for compounds **3** and **4**.

Position	3 ^b^ (*δ*_C_, Type)	3 ^b^ (*δ*_H_, Type)	4 ^a^ (*δ*_C_, Type)	4 ^a^ (*δ*_H_, Type)
2	80.2, CH	4.24, q (6.4)	78.8, CH	4.08, q (6.4)
3	86.3, C		83.9, C	
4	78.1, CH	3.94, s	78.1, CH	3.79, s
5	84.9, C		86.3, C	
6	204.7, C		91.2, CH	3.69, s
7	82.3, C		73.3, C	
8	139.1, CH	6.05, d (15.5)	140.9, CH	6.01, d (15.4)
9	129.1, CH	6.27, dd (15.5, 10.5)	128.5, CH	6.43, dd (11.0, 15.4)
10	137.3, CH	6.43, dd (15.0, 10.5)	138.1, CH	6.65, dd (11.0, 15.0)
11	132.5, CH	6.36, dd (15.0, 10.5)	131.5, CH	6.50, dd (11.0, 15.0)
12	135.8, CH	7.16, dd (15.0, 10.9)	136.1, CH	7.16, dd (11.0, 15.0)
13	119.6, CH	6.34, d (15.0)	119.4, CH	6.59, d (15.0)
14	154.5, C		154.9, C	
16	163.9, C		165.4, C	
17	88.9, CH	5.49, s	88.1, CH	5.64, s
18	170.8, C		172.1, C	
19	108.2, C		108.8, C	
20	13.1, CH_3_	1.23, d (6.5)	12.8, CH_3_	1.20, d (6.4)
21	16.9, CH_3_	1.35, s	12.8, CH_3_	1.22, s
22	12.5, CH_3_	1.39, s	26.3, CH_3_	1.30, s
23	31.4, CH_3_	1.46, s	12.2, CH_3_	1.32, s
24	9.0, CH_3_	1.96, s	7.9, CH_3_	2.02, s
25	56.3, CH_3_	3.82, s	56.3, CH_3_	3.92, s

^a^ Measure in MeOD-d_4_; ^b^ measure in CDCl_3_.

**Table 3:** Inhibitory activities against LPS-induced NO production.

Compound	IC_50_ (μM)	Inhibition Ratio at 50 μM
**1**	-	<50%
**2**	12	97%
**3**	-	<50%
**4**	-	<50%
Indometacin ^a^	35.8 ± 5.7	-

^a^ Indometacin was used as positive control for the test.

## Supplementary Materials

The HRESIMS and NMR spectrum are available online at: https://www.mdpi.com/article/10.3390/md20090583/s1. Figure S1. HRESIMS of compound **1**. Figure S2. ^1^H NMR spectrum of compound **1**. Figure S3. ^13^C NMR spectrum of compound **1**. Figure S4. HMQC spectrum of compound **1**. Figure S5. HMBC spectrum of compound **1**. Figure S6. ^1^H, ^1^H-COSY spectrum of compound **1**. Figure S7. NOESY spectrum of compound **1**. Figure S8. HRESIMS of compound **2**. Figure S9. ^1^H NMR spectrum of compound **2**. Figure S10.^13^C NMR spectrum of compound **2**. Figure S11. HMQC spectrum of compound **2**. Figure S12. HMBC spectrum of compound **2**. Figure S13. ^1^H, ^1^H-COSY spectrum of compound **2**. Figure S14. NOESY spectrum of compound **2**. Figure S15. HR-ESI-MS spectrum of compound **3**. Figure S16. ^1^H NMR spectrum of compound **3**. Figure S17. ^13^C NMR spectrum of compound **3**. Figure S18. HMQC spectrum of compound **3**. Figure S19. HMBC spectrum of compound **3**. Figure S20. ^1^H, ^1^H-COSY spectrum of compound **3**. Figure S21. NOESY spectrum of compound **3**. Figure S22. HR-ESI-MS spectrum of compound **4**. Figure S23. ^1^H NMR spectrum of compound **4**. Figure S24. ^13^C NMR spectrum of compound **4**. Figure S25. HMQC spectrum of compound **4**. Figure S26. HMBC spectrum of compound **4**. Figure S27. ^1^H, ^1^H-COSY spectrum of compound **4**. Figure S28. NOESY spectrum of compound **4**. Figure S29. Experimental (Exp.) and calculated (Cal.) ^1^H and ^13^C chemical shift values of **3** and its possible isomers. Figure S30. DP4+ analysis of **3**. Figure S31. Experimental (Exp.) and calculated (Cal.) ^1^H and ^13^C chemical shift values of **4** and its possible isomers. Figure S32. DP4+ analysis of **4**. Figure S33. Proposed biosynthetic pathways for compounds **1**–**4**.
