# Supplementary material for: New Polyketides from Mangrove Endophytic Fungus Penicillium sp. BJR-P2 and Their Anti-Inflammatory Activity
Source: Mar Drugs. 2022 Sep 18;20(9):583. doi: 10.3390/md20090583 (PMC9502265; doi:10.3390/md20090583)
Supplement: Supplementary file 1 [file marinedrugs-20-00583-s001.zip › marinedrugs-1911847-supplementary.pdf]

Supporting information

New Polyketides from Mangrove Endophytic Fungus *Penicillium* sp. BJR-P2 and their Anti-inflammatory Activity

Chen Chen, Geting Ye, Jing Tang, Jialin Li, Wenbin Liu, Li Wu, Yuhua Long*

GDMPA Key Laboratory for Process Control and Quality Evaluation of Chiral Pharmaceuticals, Guangzhou Key Laboratory of Analytical Chemistry for Biomedicine, School of Chemistry, South China Normal University, Guangzhou 510006, China

Content

Figure S1 HRESIMS of compound **1**
Figure S2 ^{1}H NMR spectrum (600 MHz, methanol-d_4) of compound **1**
Figure S3 ^{13}C NMR spectrum (600 MHz, methanol-d_4) of compound **1**
Figure S4 HMQC spectrum (600 MHz, methanol-d_4) of compound **1**
Figure S5 HMBC spectrum (600 MHz, methanol-d_4) of compound **1**
Figure S6 ^{1}H-^{1}H COSY spectrum (600 MHz, methanol-d_4) of compound **1**
Figure S7 NOESY spectrum (600 MHz, methanol-d_4) of compound **1**
Figure S8 HRESIMS of compound **2**
Figure S9 ^{1}H NMR spectrum (600 MHz, chloroform -*d*) of compound **2**
Figure S10 ^{13}C NMR spectrum (600 MHz, chloroform -*d*) of compound **2**
Figure S11 HMQC spectrum (600 MHz, chloroform -*d*) of compound **2**
Figure S12 HMBC spectrum (600 MHz, chloroform -*d*) of compound **2**
Figure S13 ^{1}H-^{1}H COSY spectrum (600 MHz, chloroform -*d*) of compound **2**
Figure S14 NOESY spectrum (600 MHz, chloroform -*d*) of compound **2**
Figure S15 HRESIMS of compound **3**
Figure S16 ^{1}H NMR spectrum (600 MHz, chloroform -*d*) of compound **3**
Figure S17 ^{13}C NMR spectrum (600 MHz, chloroform -*d*) of compound **3**
Figure S18 HMQC spectrum (600 MHz, chloroform -*d*) of compound **3**
Figure S19 HMBC spectrum (600 MHz, chloroform -*d*) of compound **3**
Figure S20 ^{1}H-^{1}H COSY spectrum (600 MHz, chloroform -*d*) of compound **3**
Figure S21 NOESY spectrum (600 MHz, chloroform -*d*) of compound **3**
Figure S22 HRESIMS of compound **4**
Figure S23 ^{1}H NMR spectrum (600 MHz, methanol-d_4) of compound **4**
Figure S24 ^{13}C NMR spectrum (600 MHz, methanol-d_4) of compound **4**
Figure S25 HMQC spectrum (600 MHz, methanol-d_4) of compound **4**
Figure S26 HMBC spectrum (600 MHz, methanol-d_4) of compound **4**
Figure S27 ^{1}H-^{1}H COSY spectrum (600 MHz, methanol-d_4) of compound **4**
Figure S28 NOESY spectrum (600 MHz, methanol-d_4) of compound **4**
Figure S29 Experimental (Exp.) and calculated (Cal.) ^{1}H and ^{13}C chemical shift

values of **3** and its possible isomers
Figure S30 DP4+ analysis of **3**
Figure S31 Experimental (Exp.) and calculated (Cal.) ^{1}H and ^{13}C chemical shift values of **4** and its possible isomers
Figure S32 DP4+ analysis of **4**
Figure S33 Proposed biosynthetic pathways for compounds **1- 4.**

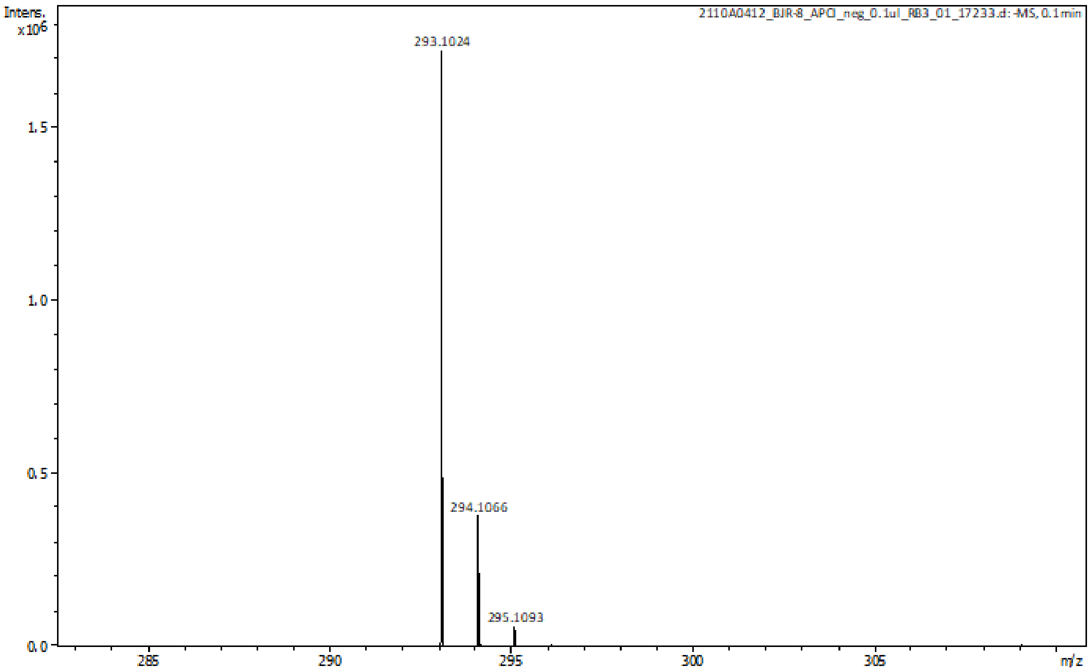

SPECTRUM - simulation :

m/z	Theo. Mass	Delta (ppm)	RDB equiv.	Composition
293.1024	293.1031	2.3	7	$C_{15}H_{17}O_6$

Figure S1 HRESIMS of compound **1**

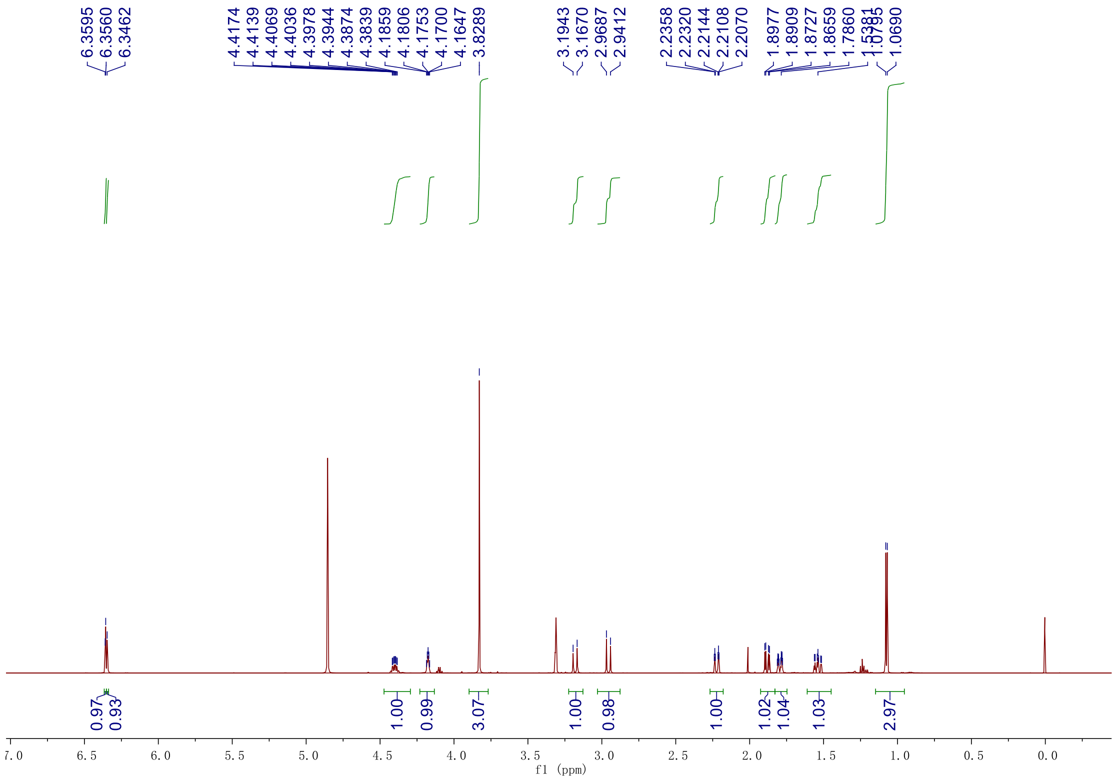

Figure S2 ^{1}H NMR spectrum (600 MHz, methanol-*d*$_4$) of compound **1**

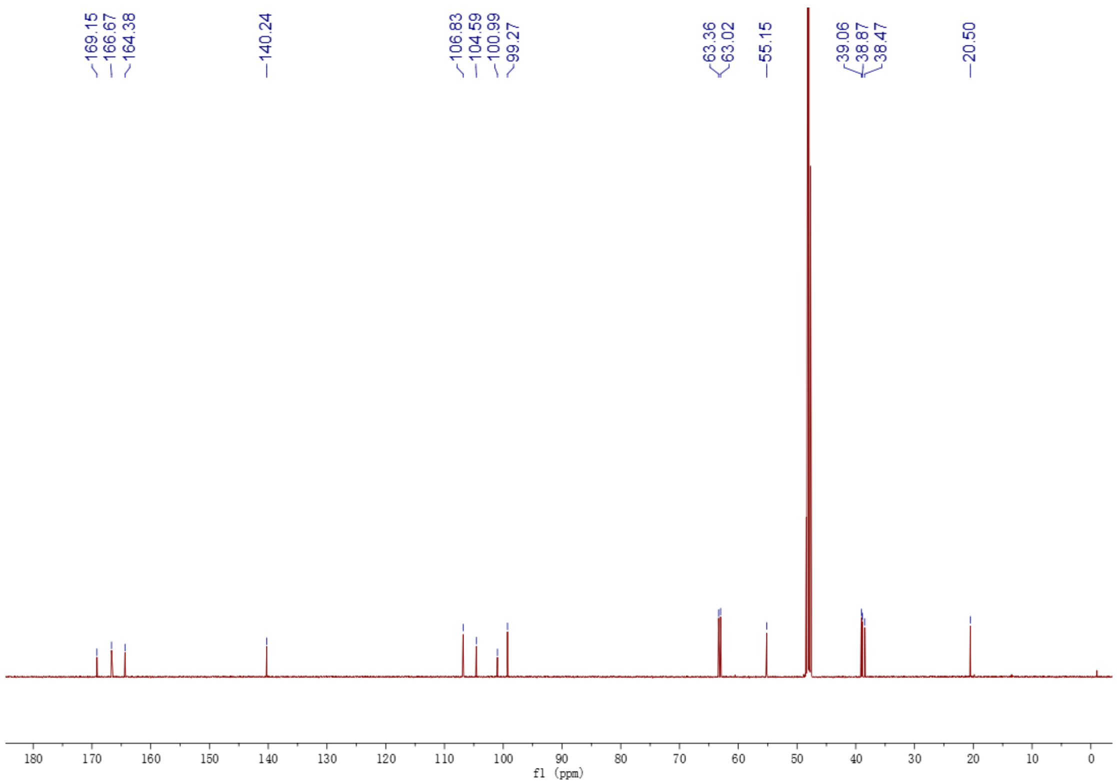

Figure S3 ^{13}C NMR spectrum (600 MHz, methanol-d_4) of compound **1**

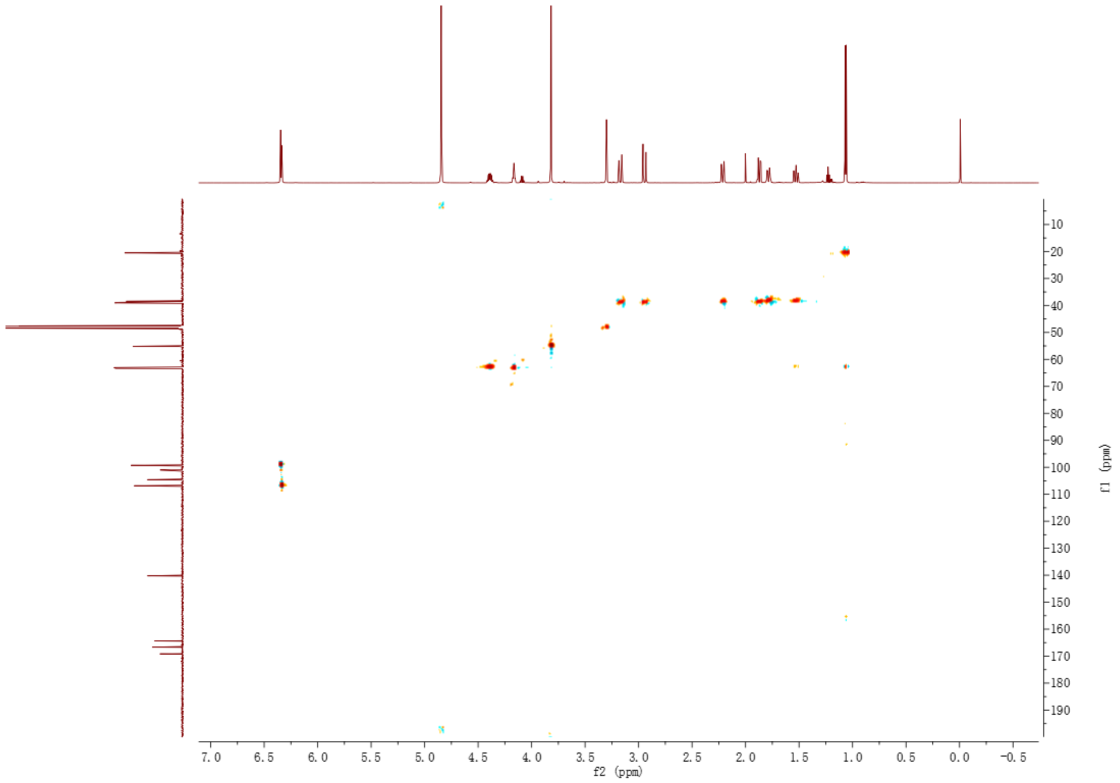

Figure S4 HMQC spectrum (600 MHz, methanol-d_4) of compound **1**

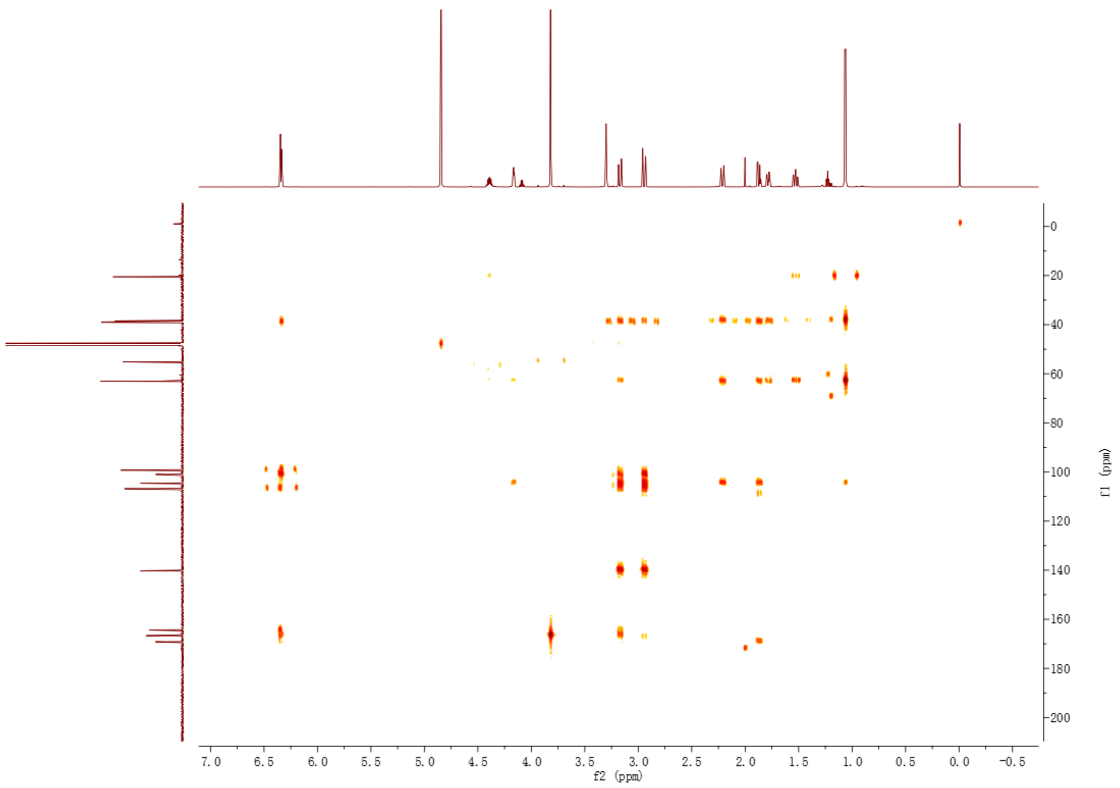

Figure S5 HMBC spectrum (600 MHz, methanol-d_4) of compound **1**

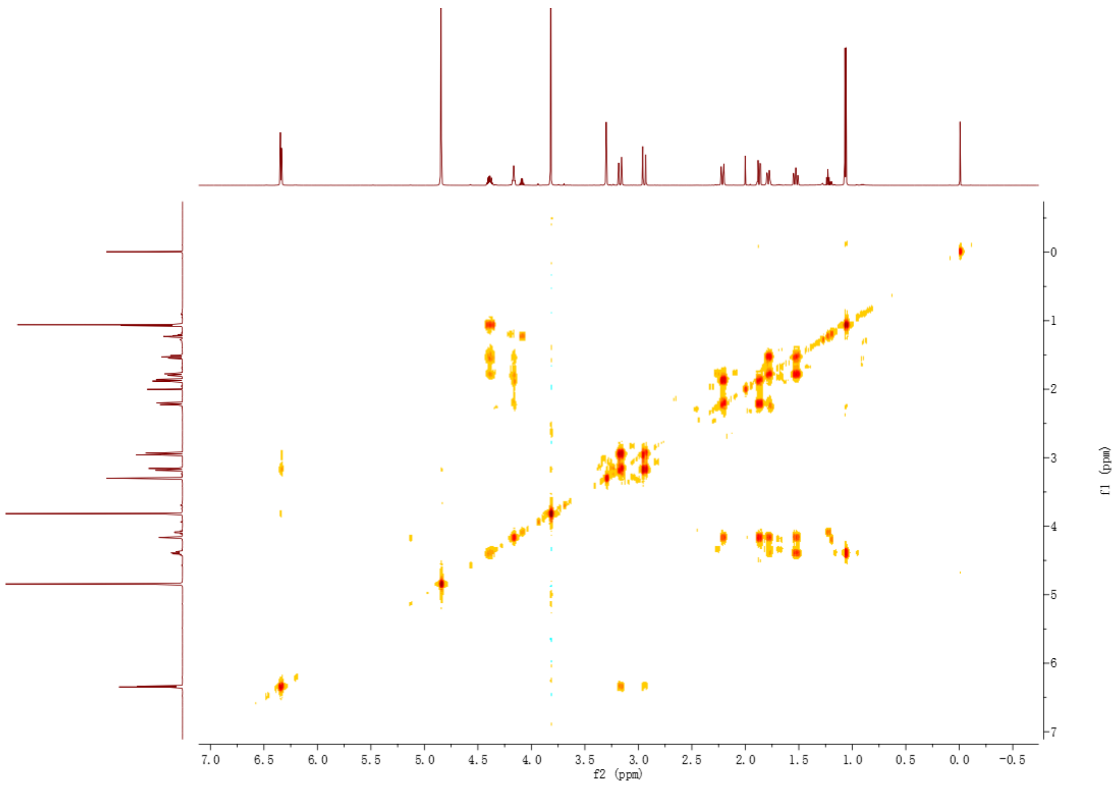

Figure S6 ^{1}H-^{1}H COSY spectrum (600 MHz, methanol-d_4) of compound **1**

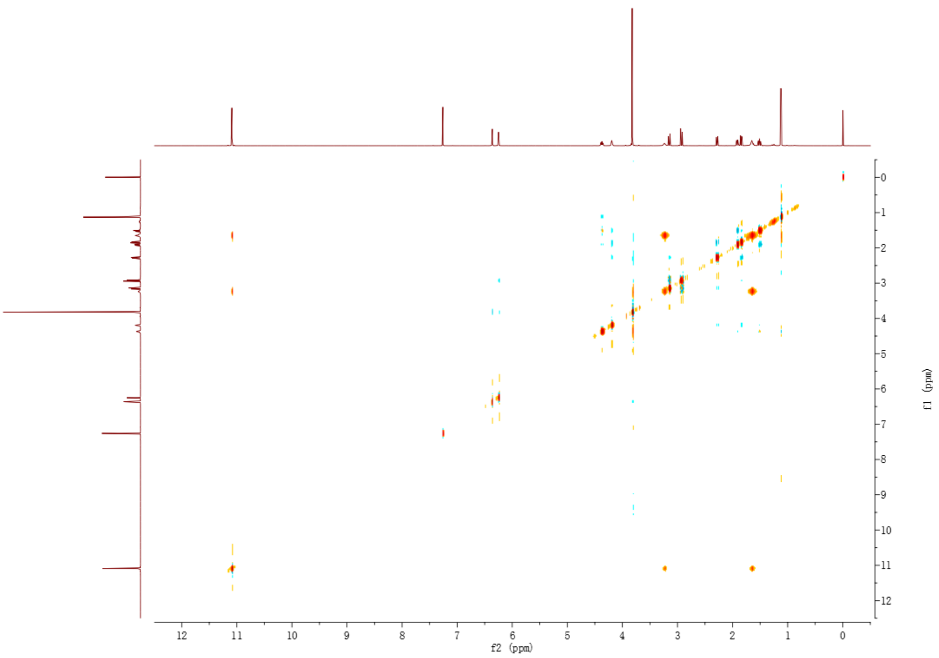

Figure S7 NOESY spectrum (600 MHz, chloroform -*d*) of compound **1**

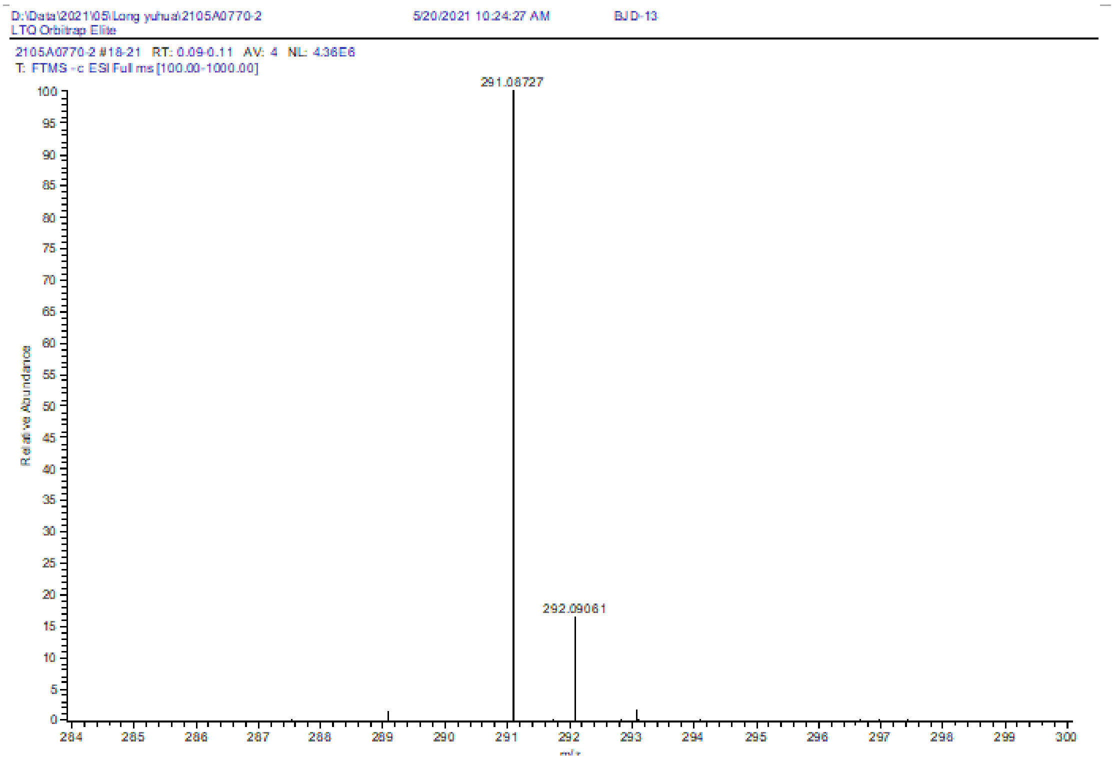

SPECTRUM - simulation :

m/z	Theo. Mass	Delta (ppm)	RDB equiv.	Composition
291.08727	291.08741	-0.14	8.5	C_{15} H_{15} O_6

Figure S8 HRESIMS of compound **2**

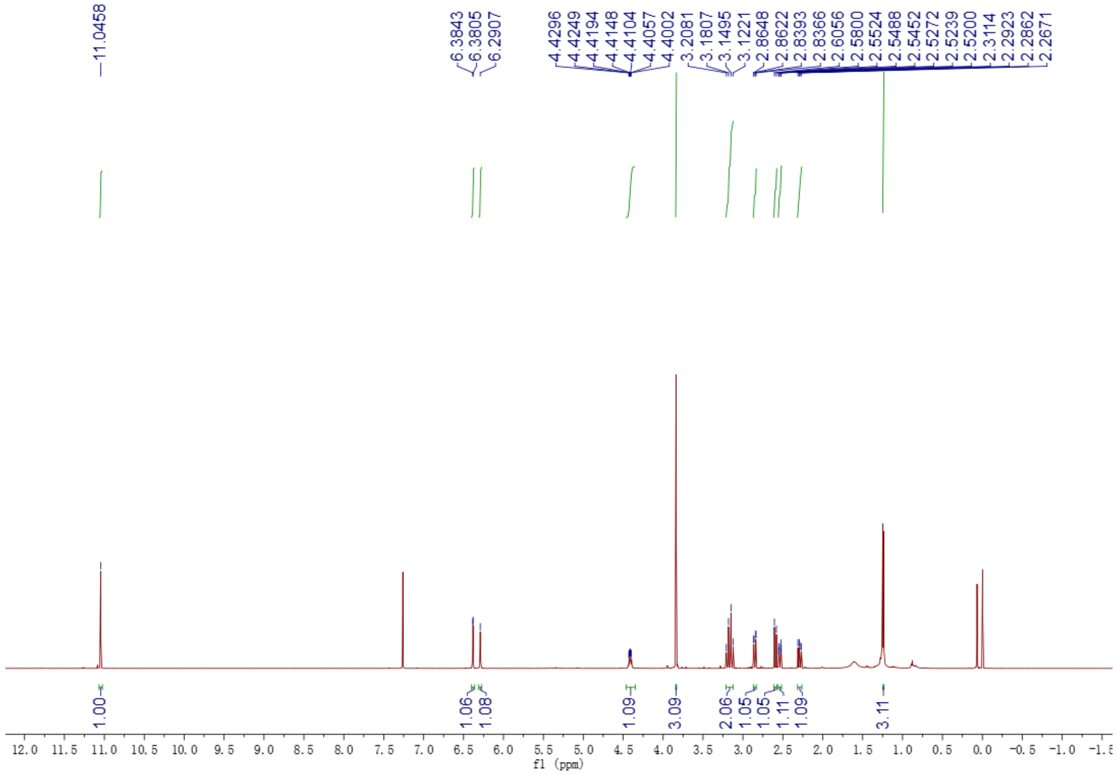

Figure S9 ^{1}H NMR spectrum (600 MHz, chloroform -*d*) of compound **2**

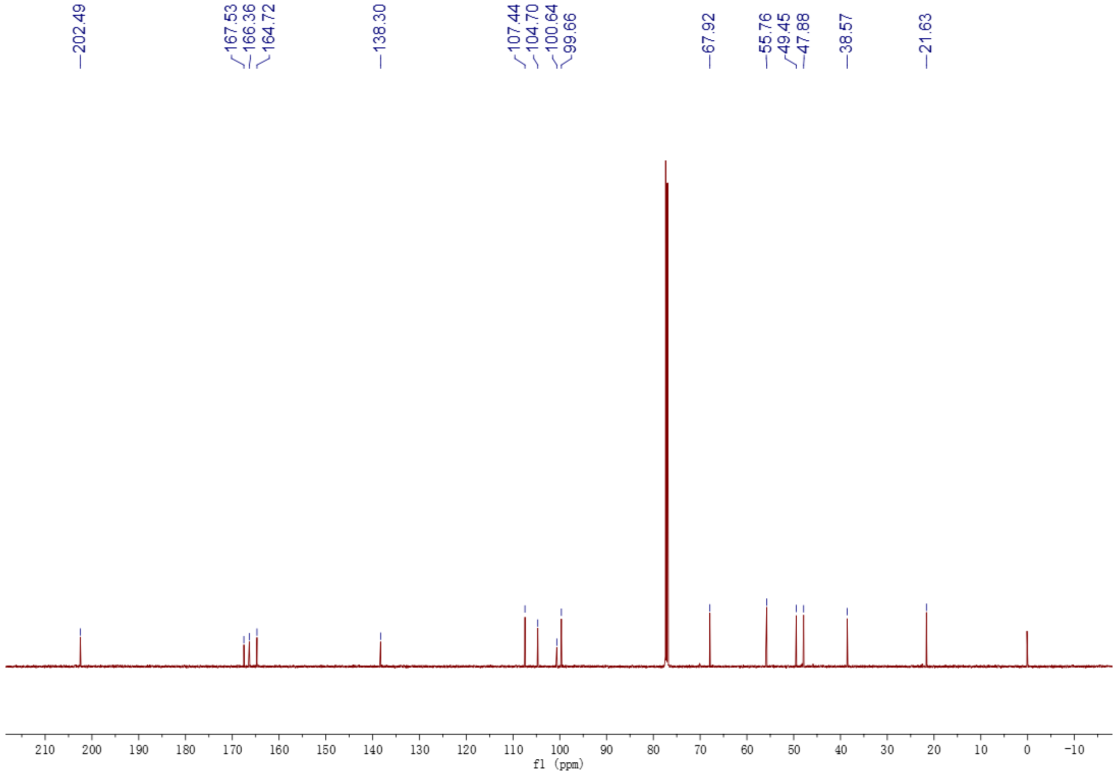

Figure S10 ^{13}C NMR spectrum (600 MHz, chloroform -*d*) of compound **2**

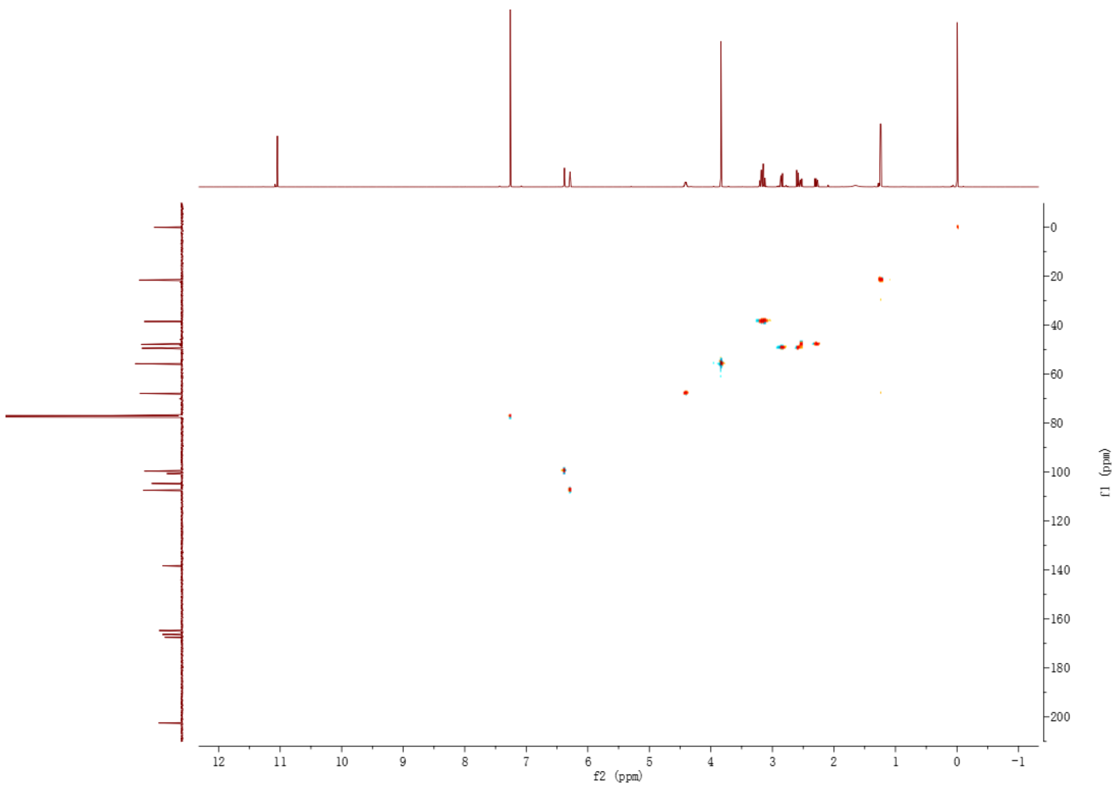

Figure S11 HMQC spectrum (600 MHz, chloroform -*d*) of compound **2**

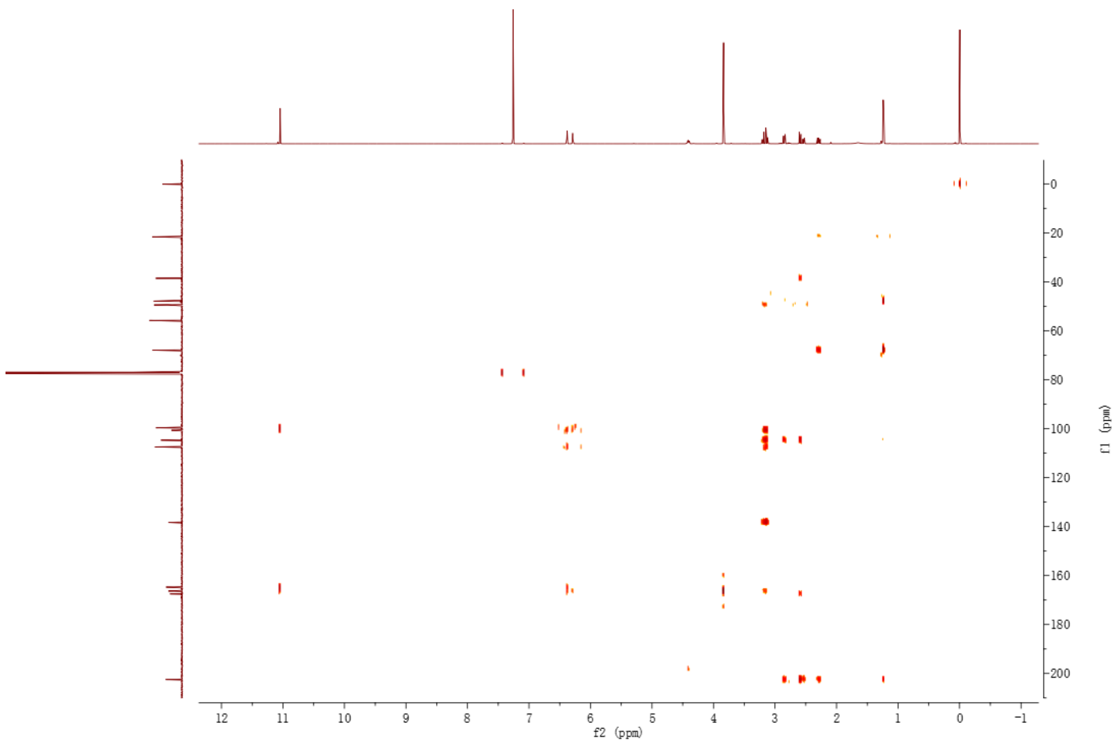

Figure S12 HMBC spectrum (600 MHz, chloroform -*d*) of compound **2**

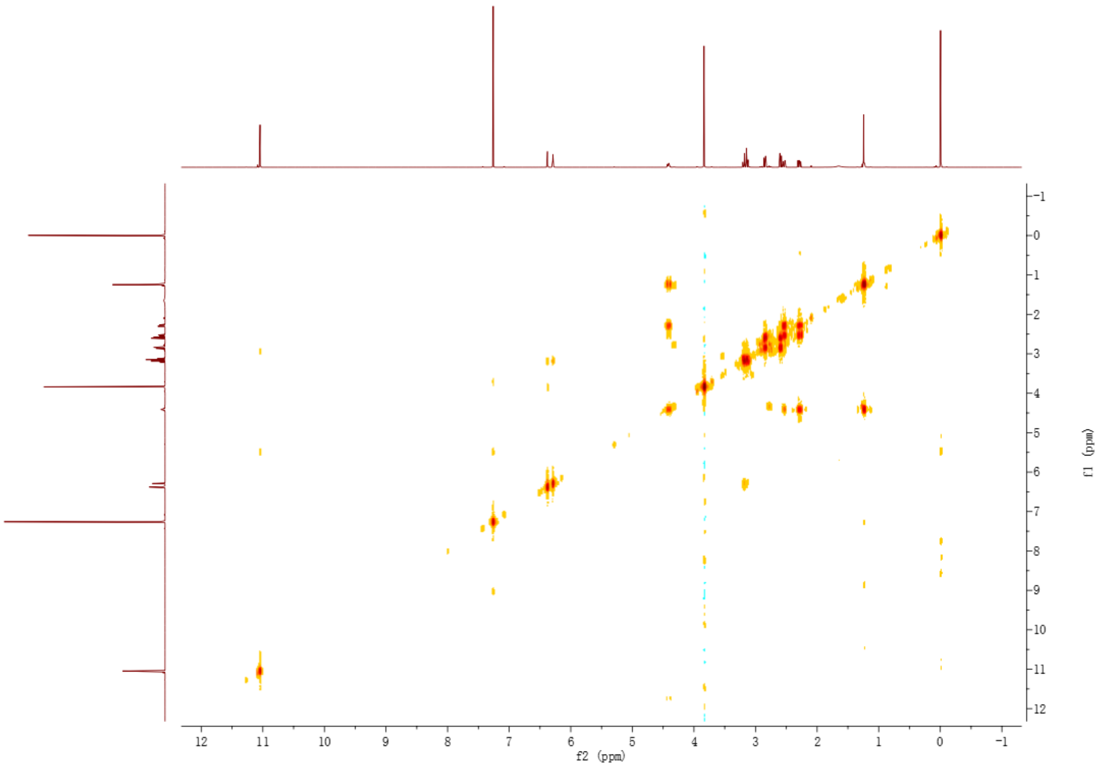

Figure S13 ^{1}H-^{1}H COSY spectrum (600 MHz, chloroform -*d*) of compound **2**

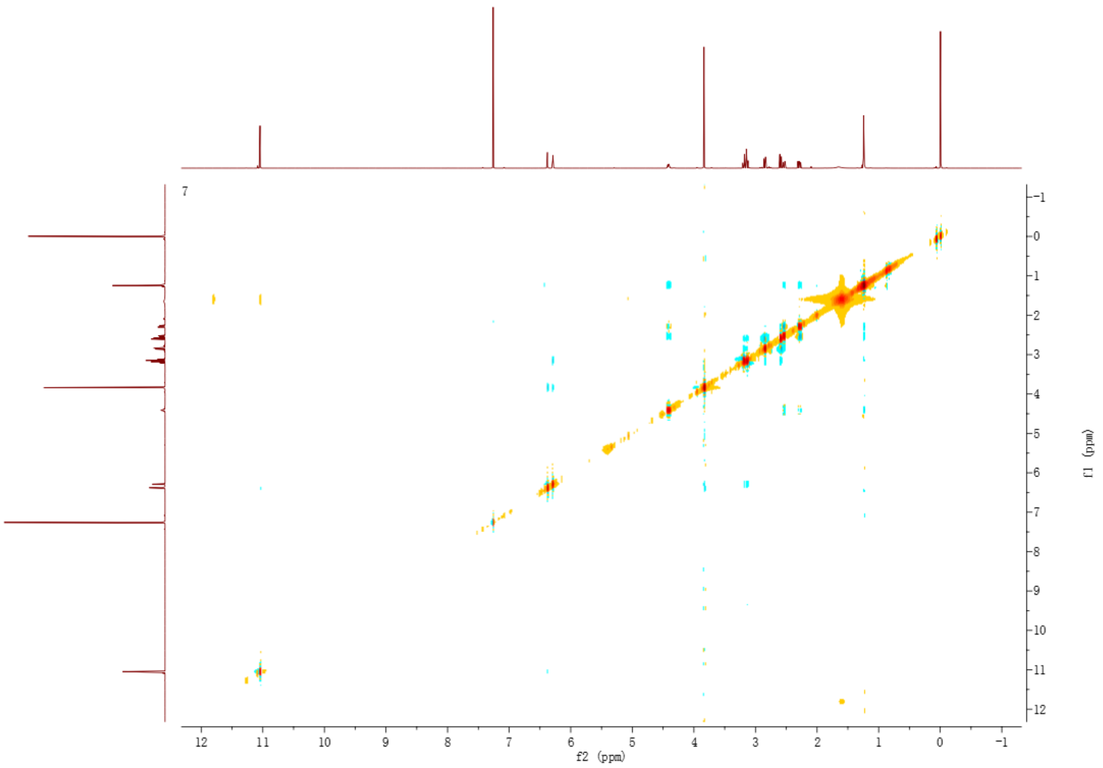

Figure S14 NOESY spectrum (600 MHz, chloroform -*d*) of compound **2**

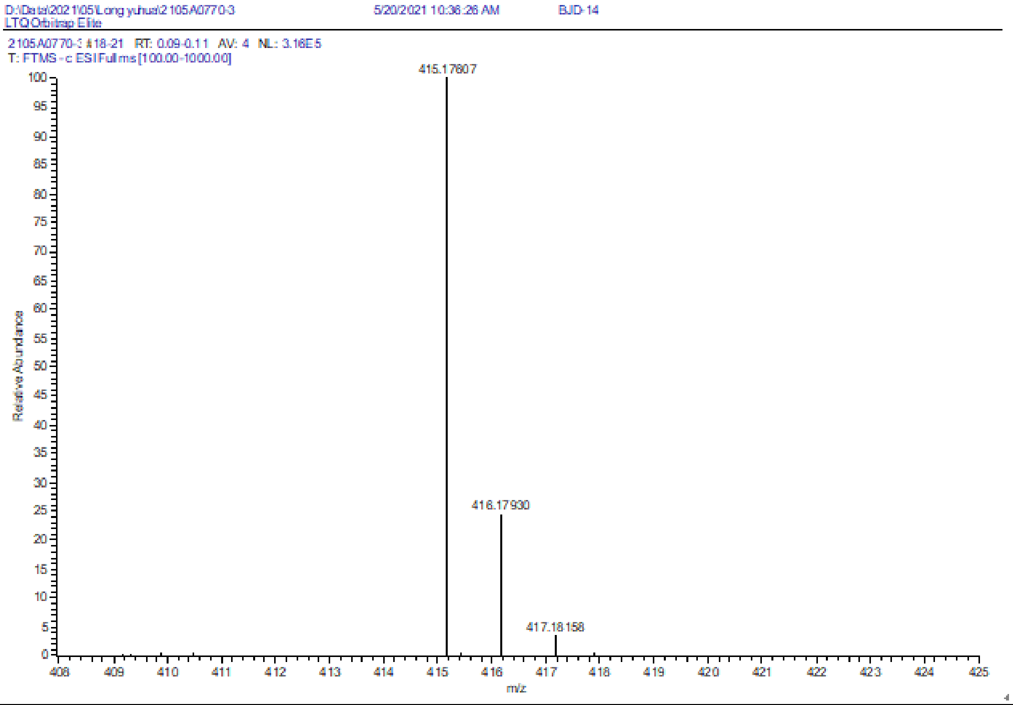

SPECTRUM - simulation :

m/z	Theo. Mass	Delta (ppm)	RDB equiv.	Composition
415.17607	415.17623	-0.38	10.5	$C_{23}H_{27}O_7$

Figure S15 HRESIMS of compound **3**

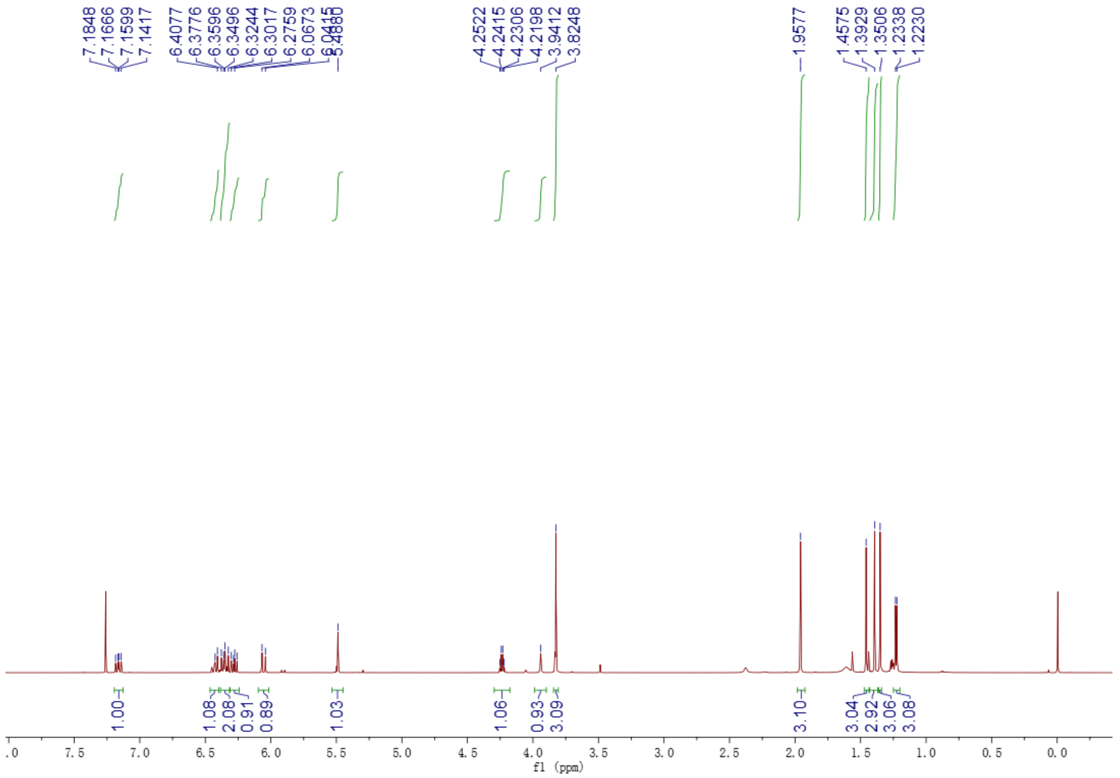

Figure S16 ^{1}H NMR spectrum (600 MHz, chloroform -*d*) of compound **3**

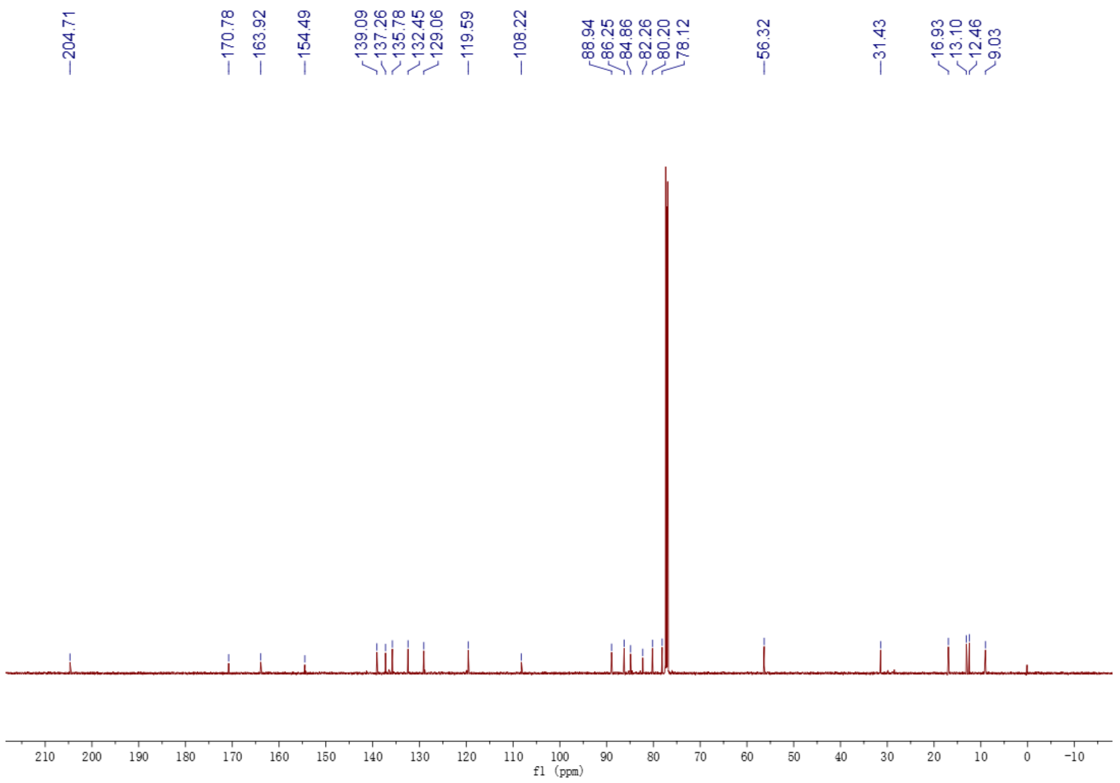

Figure S17 ^{13}C NMR spectrum (600 MHz, chloroform -*d*) of compound **3**

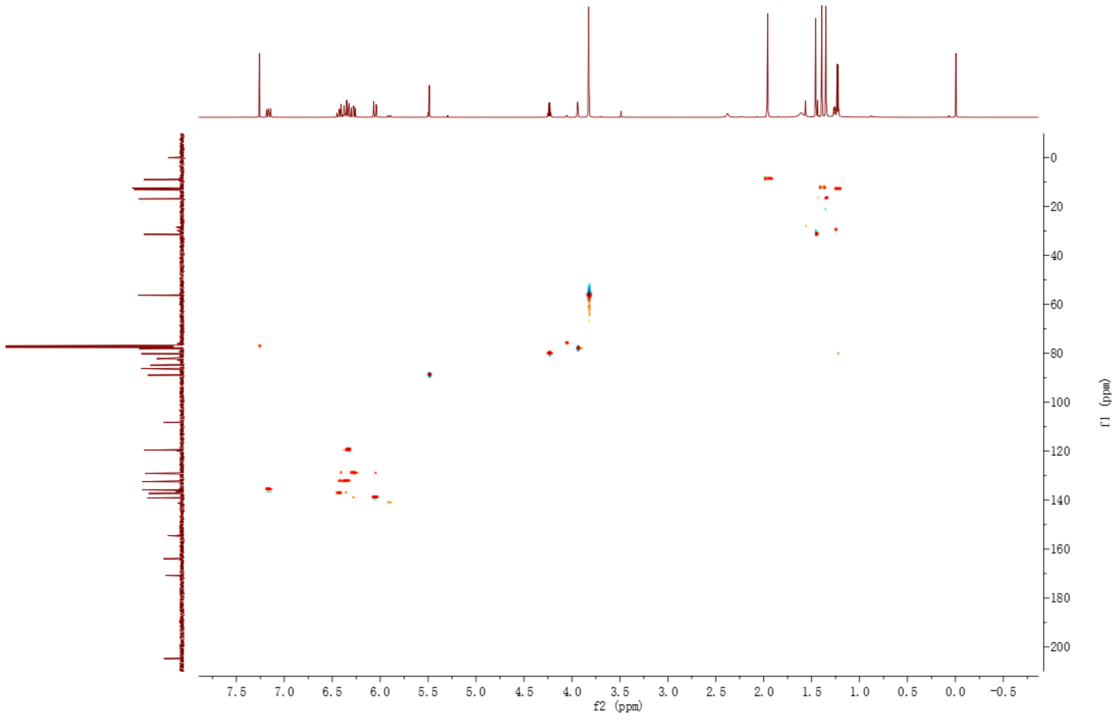

Figure S18 HMQC spectrum (600 MHz, chloroform -*d*) of compound **3**

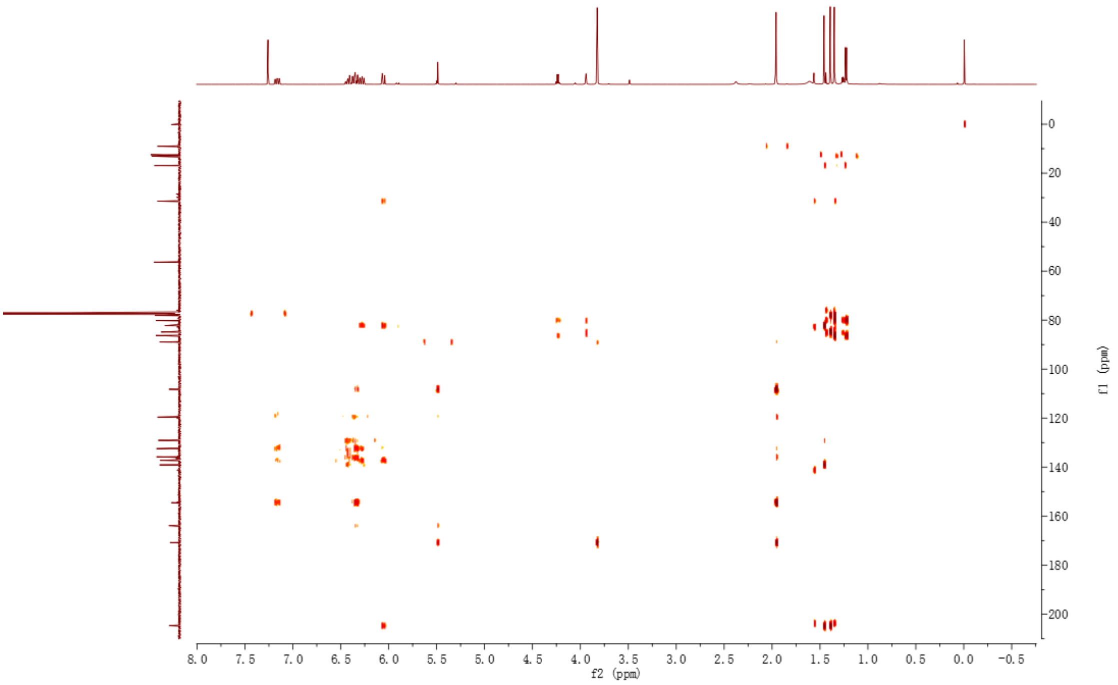

Figure S19 HMBC spectrum (600 MHz, chloroform -*d*) of compound **3**

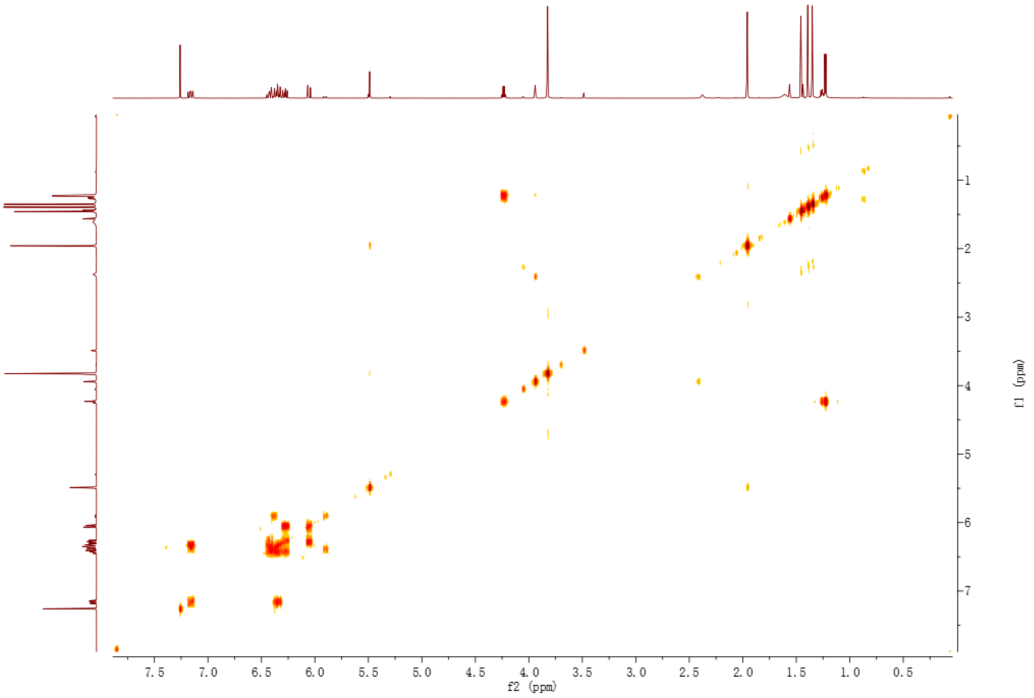

Figure S20 ^{1}H-^{1}H COSY spectrum (600 MHz, chloroform -*d*) of compound **3**

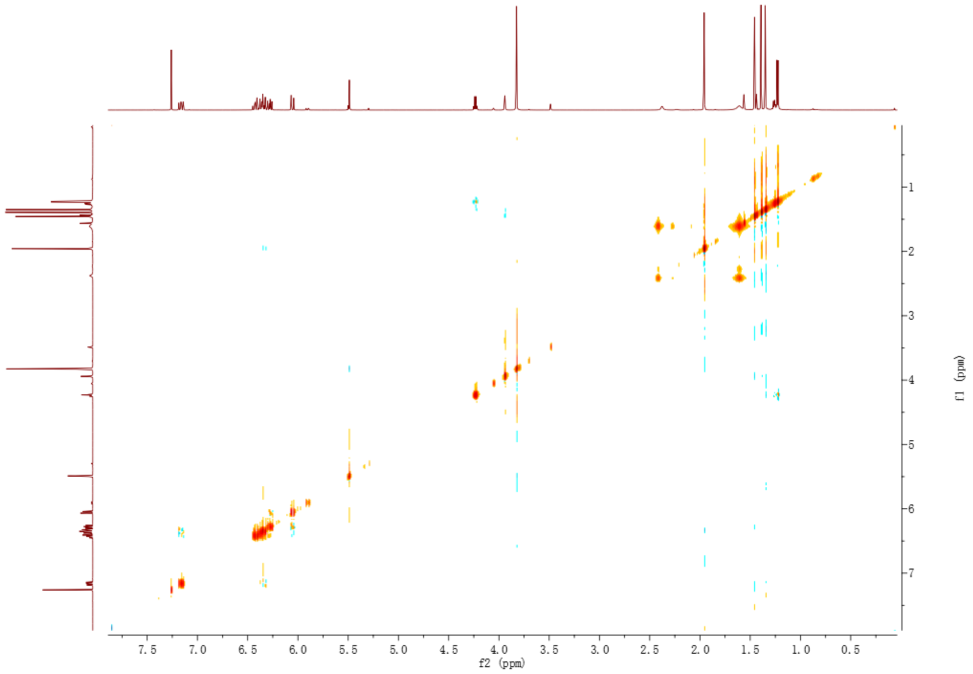

Figure S21 NOESY spectrum (600 MHz, chloroform -*d*) of compound **3**

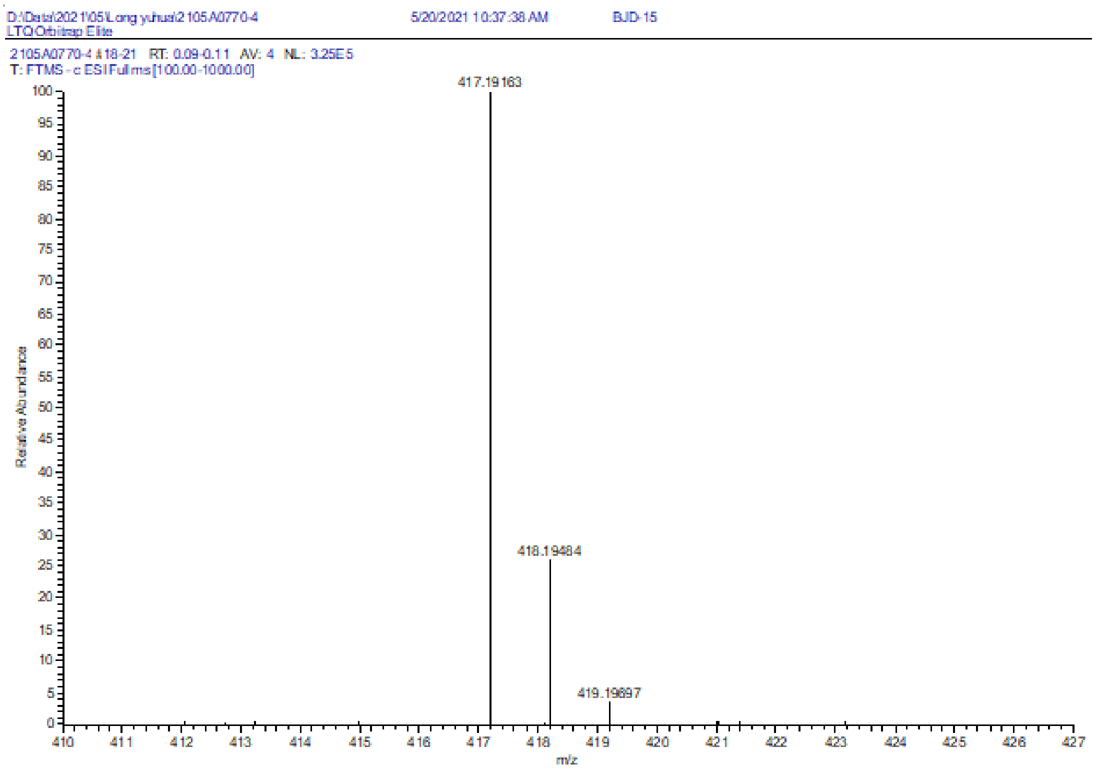

SPECTRUM - simulation :

m/z	Theo. Mass	Delta (ppm)	RDB equiv.	Composition
417.19163	417.19188	-0.59	9.5	$C_{23}H_{29}O_7$

Figure S22 HRESIMS of compound **4**

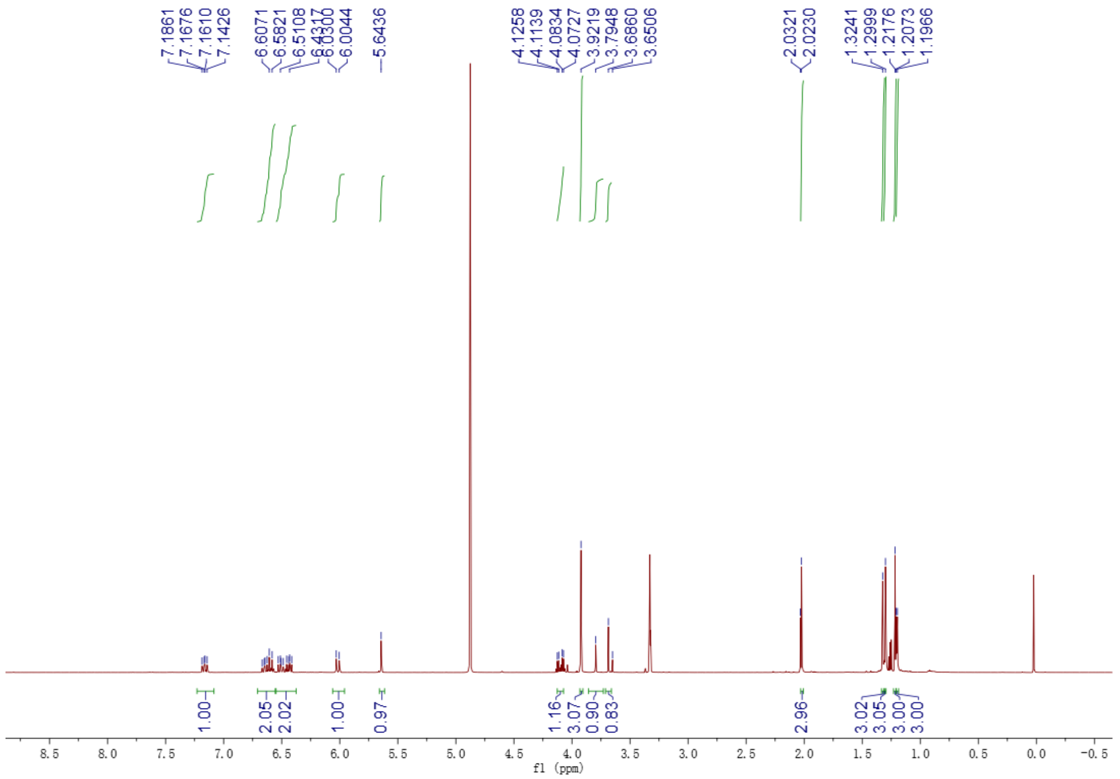

Figure S23 ^{1}H NMR spectrum (600 MHz, methanol-d_4) of compound **4**

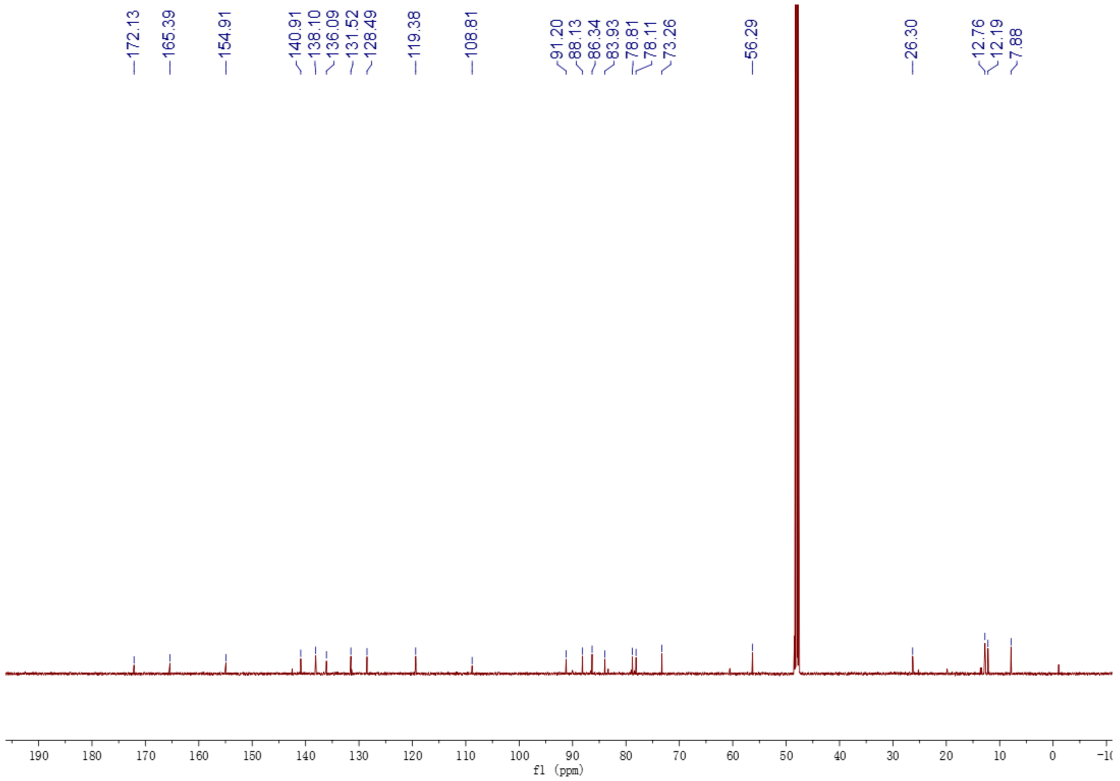

Figure S24 ^{13}C NMR spectrum (600 MHz, methanol-d_4) of compound **4**

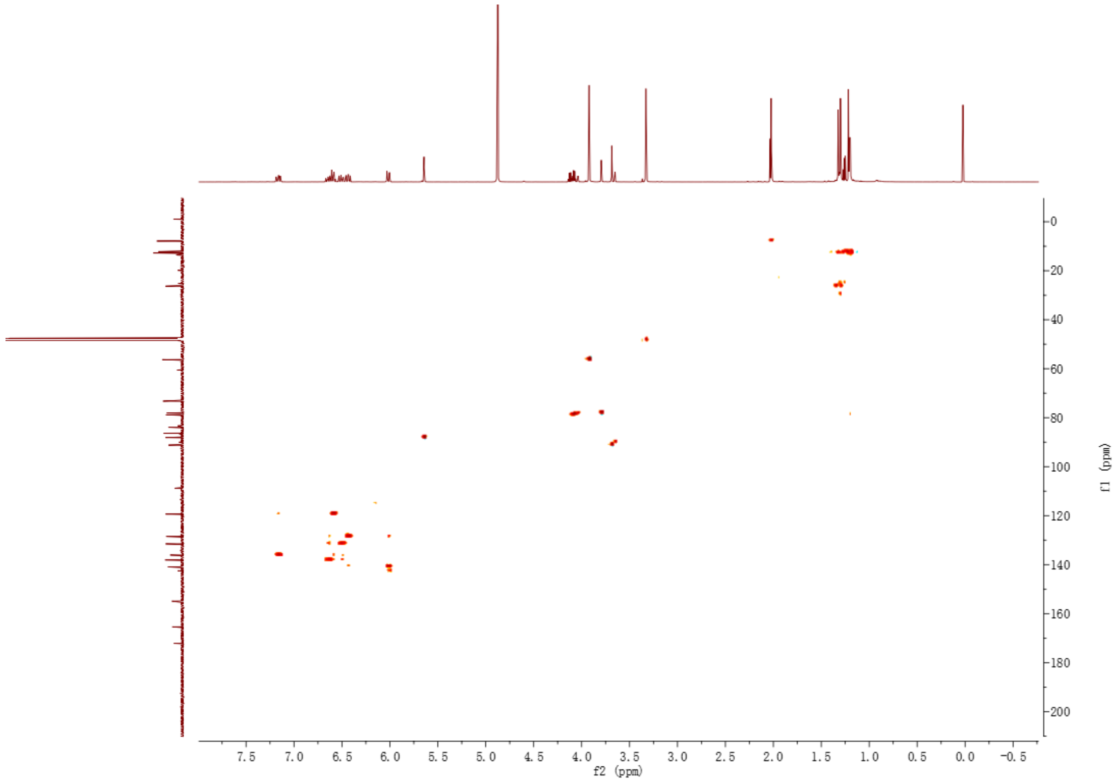

Figure S25 HMQC spectrum (600 MHz, methanol-d_4) of compound **4**

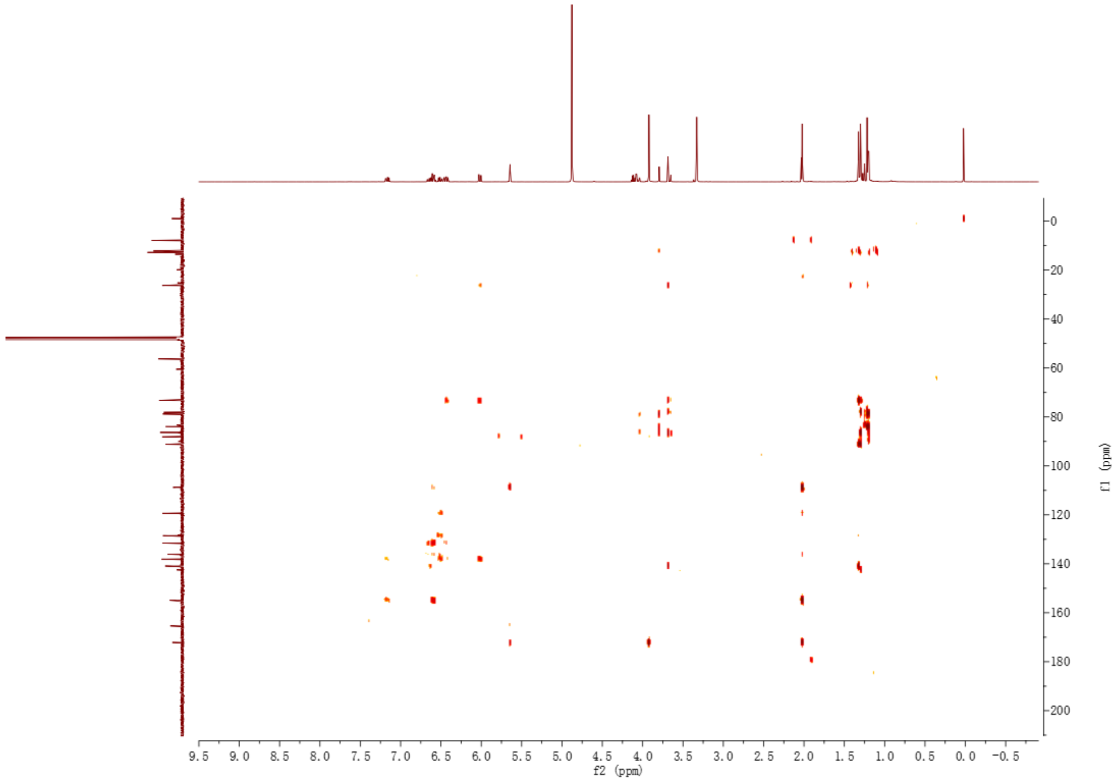

Figure S26 HMBC spectrum (600 MHz, methanol-d_4) of compound **4**

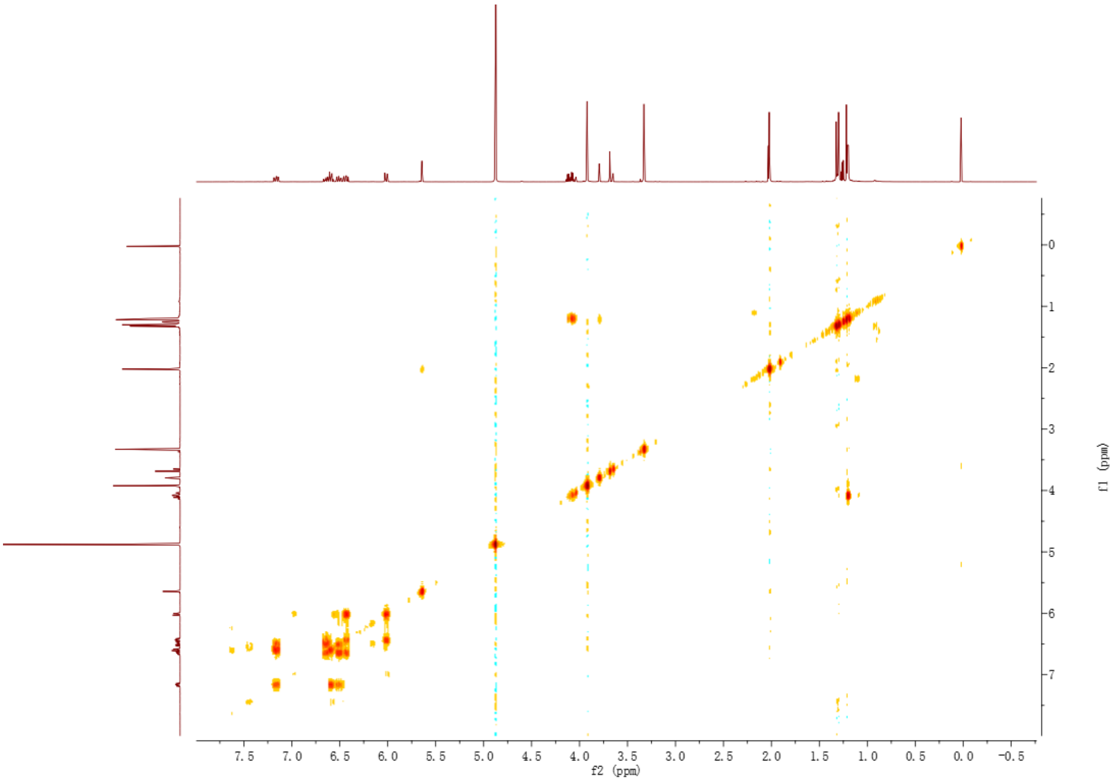

Figure S27 ^{1}H-^{1}H COSY spectrum (600 MHz, methanol-d_4) of compound **4**

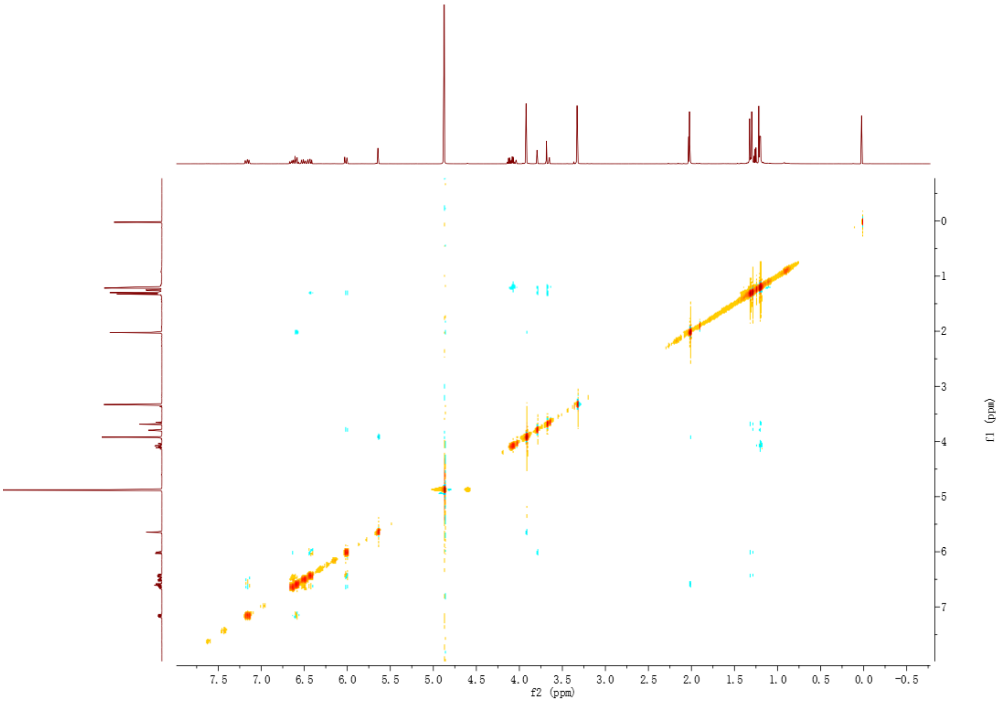

Figure S28 NOESY spectrum (600 MHz, methanol-d_4) of compound **4**

Functional	Solvent?	Basis Set	Type of Data
mPW1PW91	PCM	6-31+G(d,p)	Unscaled Shifts

		DP4+	#REF!	-	-	-	-
Nuclei	sp2?	xperimenta	Isomer 1	Isomer 2	Isomer 3	Isomer 4	Isomer 5
C		80.2	79.6	78.0			
C		86.25	79.1	80.3			
C		78.12	87.6	86.6			
C		84.86	86.9	91.7			
C		204.71	223.3	218.2			
C		82.26	85.7	83.9			
C		139.09	143.1	138.1			
C		129.06	130.0	131.8			
C		137.26	140.2	134.0			
C		132.45	132.7	128.8			
C		135.78	137.6	132.1			
C		119.59	119.34	115.48			
C		154.49	156.36	155.76			
C		108.22	110.45	111.01			
C		170.78	170.66	170.66			
C		88.94	88.54	87.70			
C		163.92	162.68	162.51			
C		12.46	23.16	22.67			
C		13.1	12.22	11.70			
C		31.43	27.36	26.86			
C		9.03	10.07	10.26			
C		56.32	55.23	55.42			
C		16.93	18.87	16.82			
H		4.24	3.83	3.78			
H		3.94	3.88	3.93			
H		6.05	6.00	5.69			
H		6.43	6.71	6.80			
H		6.27	6.79	7.17			
H		6.36	6.58	6.27			
H		7.16	7.32	7.01			
H		6.34	6.48	7.07			
H		5.49	5.32	5.29			
H		1.39	1.33	1.37			
H		1.23	1.24	1.24			
H		1.46	1.42	1.41			
H		1.96	1.96	1.97			
H		3.82	3.72	3.72			
H		1.35	1.13	1.14			

Figure S29 Experimental (Exp.) and calculated (Cal.) ^{1}H and ^{13}C chemical shift values of **3** and its possible isomers, respectively

Functional	Solvent?	Basis Set	Type of Data
mPW1PW91	PCM	6-31+G(d,p)	Unscaled Shifts

	Isomer 1	Isomer 2	Isomer 3	Isomer 4	Isomer 5	Isomer 6
sDP4+ (H data)	100.00%	0.00%	-	-	-	-
sDP4+ (C data)	99.98%	0.02%	-	-	-	-
sDP4+ (all data)	100.00%	0.00%	-	-	-	-
uDP4+ (H data)	99.86%	0.14%	-	-	-	-
uDP4+ (C data)	100.00%	0.00%	-	-	-	-
uDP4+ (all data)	100.00%	0.00%	-	-	-	-
DP4+ (H data)	100.00%	0.00%	-	-	-	-
DP4+ (C data)	100.00%	0.00%	-	-	-	-
DP4+ (all data)	100.00%	0.00%	-	-	-	-

Figure S30 DP4+ analysis of **3**

Functional<br>mPW1PW91		Solvent?<br>PCM		Basis Set<br>6-31+G(d,p)		Type of Data<br>Unscaled Shifts	
		DP4+	100.00%	0.00%	0.00%	0.00%	-
Nuclei	sp2?	xperiment	Isomer 1	Isomer 2	Isomer 3	Isomer 4	Isomer 5
C		78.81	78.6	82.9	82.9	79.4	
C		83.93	80.5	79.3	79.0	79.9	
C		78.11	80.9	75.4	74.6	81.4	
C		86.34	88.0	89.5	86.2	88.6	
C		91.2	82.1	81.4	81.3	81.7	
C		73.26	77.8	79.7	78.7	79.9	
C		140.91	147.2	145.0	151.9	150.5	
C		128.49	127.9	127.3	126.7	125.1	
C		138.1	140.7	140.6	141.2	141.0	
C		131.52	132.6	131.9	131.7	132.7	
C		136.09	137.6	137.7	143.3	142.7	
C		119.38	119.41	119.09	124.01	124.35	
C		154.91	156.46	156.68	159.47	159.73	
C		108.81	111.21	111.28	110.91	111.49	
C		172.13	171.88	171.64	171.57	170.98	
C		88.13	88.09	87.57	87.73	87.29	
C		165.39	163.87	163.90	164.14	164.23	
C		26.3	17.96	21.77	21.31	17.61	
C		12.76	12.66	15.21	15.37	12.75	
C		12.19	20.45	28.75	24.26	22.50	
C		7.88	10.35	10.28	11.78	11.72	
C		56.29	56.06	55.81	55.83	55.56	
C		12.76	20.88	23.53	23.47	21.66	
H		4.08	3.79	3.97	3.92	3.74	
H		3.79	3.79	4.38	4.12	3.70	
H		3.69	3.59	3.51	3.46	3.53	
H		6.01	6.38	6.03	6.31	6.46	
H		6.43	6.37	6.53	6.53	6.31	
H		6.65	6.65	6.65	6.68	6.65	
H		6.5	6.52	6.50	6.41	6.45	
H		7.16	7.23	7.23	6.87	6.80	
H		6.59	6.46	6.45	6.21	6.19	
H		5.64	5.33	5.38	5.30	5.31	
H		1.3	1.25	1.28	1.51	1.37	
H		1.2	1.19	1.27	1.19	1.19	
H		1.32	1.38	1.49	1.34	1.37	
H		2.02	1.90	1.89	2.10	2.13	
H		3.92	3.74	3.74	3.72	3.74	
H		1.22	1.18	1.26	1.26	1.15	

Figure S31 Experimental (Exp.) and calculated (Cal.) ^{1}H and ^{13}C chemical shift values of **4** and its possible isomers, respectively

Functional<br>mPW1PW91	Solvent?<br>PCM		Basis Set<br>6-31+G(d,p)		Type of Data<br>Unscaled Shifts	
	Isomer 1	Isomer 2	Isomer 3	Isomer 4	Isomer 5	Isomer 6
sDP4+ (H data)	96.69%	2.92%	0.01%	0.39%	-	-
sDP4+ (C data)	100.00%	0.00%	0.00%	0.00%	-	-
sDP4+ (all data)	100.00%	0.00%	0.00%	0.00%	-	-
uDP4+ (H data)	93.49%	6.47%	0.01%	0.04%	-	-
uDP4+ (C data)	99.94%	0.05%	0.00%	0.00%	-	-
uDP4+ (all data)	100.00%	0.00%	0.00%	0.00%	-	-
DP4+ (H data)	99.79%	0.21%	0.00%	0.00%	-	-
DP4+ (C data)	100.00%	0.00%	0.00%	0.00%	-	-
DP4+ (all data)	100.00%	0.00%	0.00%	0.00%	-	-

Figure S32 DP4+ analysis of **4**

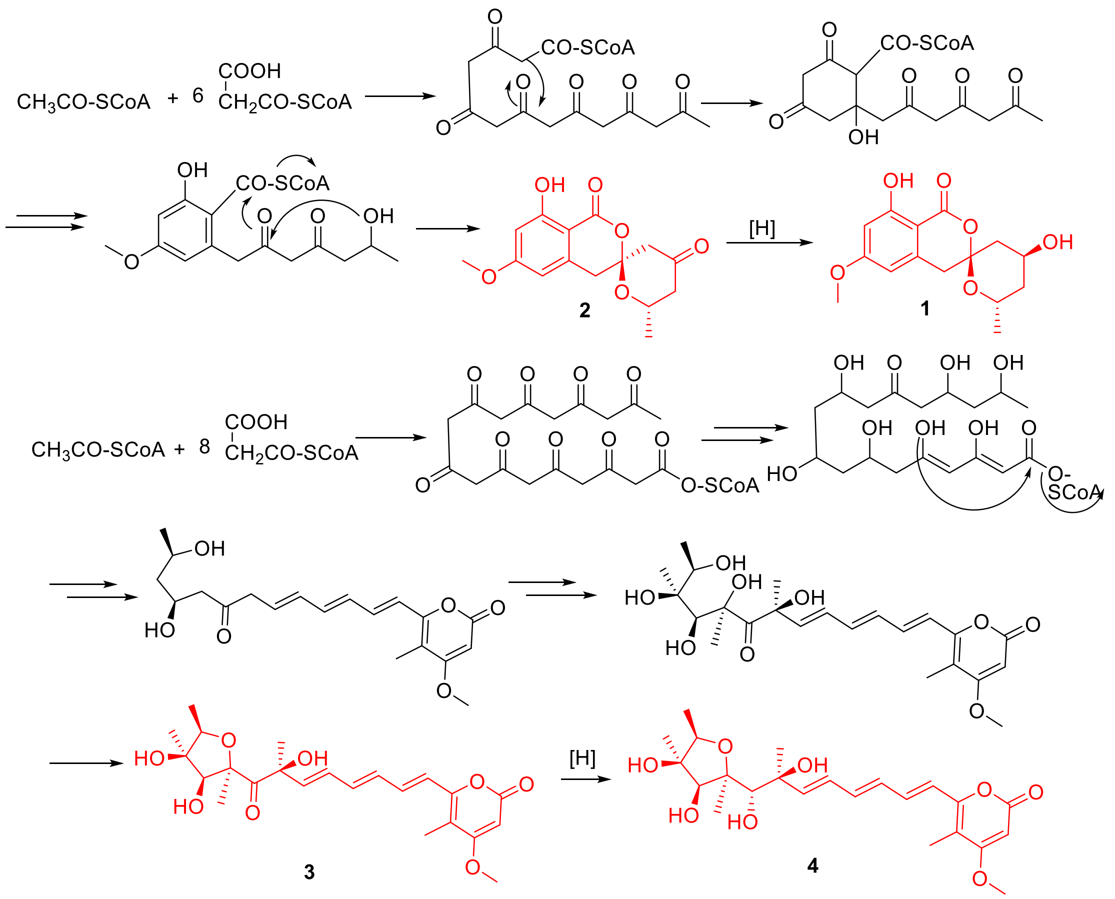

Figure S33 Proposed biosynthetic pathways for compounds **1- 4.**
